# Convergent Valorization via Scalable C═N Bond Electrosynthesis Through Metal‐Dopant Interfacial Engineering in a Flow Electrolyzer

**DOI:** 10.1002/anie.9964729

**Published:** 2026-03-30

**Authors:** Ruijie Yi, Jiu Chen, Xiaoyong Mo, Tian Zeng, Jingtao Zhou, Fulai Liu, Shu‐Chih Haw, Yong Chen, Ruosong Li, Edmund C. M. Tse

**Affiliations:** ^1^ Department of Chemistry, HKU‐CAS Joint Laboratory on New Materials University of Hong Kong Hong Kong Hong Kong SAR China; ^2^ School of Chemical Engineering Northwest University Xi'an Shaanxi China; ^3^ Key Laboratory of Photochemical Conversion and Optoelectronic Materials & CAS‐HKU Joint Laboratory on New Materials, Technical Institute of Physics and Chemistry Chinese Academy of Sciences Beijing P. R. China; ^4^ University of Chinese Academy of Sciences Beijing P. R. China; ^5^ National Synchrotron Radiation Research Center (NSRRC) Hsinchu Science Park Hsinchu Taiwan

**Keywords:** C═N bond formation, electrosynthesis, formaldoxime, metal doping, scalability

## Abstract

Electrocatalytic conversion of C‐ and N‐containing pollutants into value‐added C–N‐bond containing compounds has attracted increasing attention. Specifically, the exploration centers on the generation of oximes featuring C═N bonds, which are essential chemicals with widespread applications in the pharmaceutical and fine chemical fields. Here, we develop an integrated electrocatalytic platform to produce formaldoxime as the target product in a green solvent using metal‐doped MoS_2_ as efficient catalysts. Notably, Fe‐doped MoS_2_ significantly boosts the Faradaic efficiency and yield rate for H_2_C═NOH to 81.2% and 963 mmol h^−1^ g^−1^, respectively. Mechanistic studies reveal that the Fe dopants enhance the NO_2_^–^ bonding, promoting substrate engagement and subsequently H_2_C═NOH formation in an aqueous medium. Upon incorporating into a flow electrolyzer, the yield rate for H_2_C═NOH electrosynthesis is drastically enhanced to 2630 mmol h^−1^ g^−1^, almost triple that obtained from H‐cell setups. Our techno‐economic analysis estimates that the daily profit of this dual‐upgrading technology reaches $230 000+, highlighting the translational advantage of our strategy. Overall, this work establishes a non‐precious metal‐dopant strategy that upcycles low‐cost C‐ and N‐containing pollutants into valuable organonitrogens, enabling renewable energy synthesis of more valuable and functionally diverse commodities.

## Graphical abstract

This study establishes an innovative approach to synthesize organonitrogens featuring C═N bonds efficiently using non‐precious metal‐doped MoS_2_ in a flow electrolyzer. Fe dopant significantly enhances Faradaic efficiency and yield rate, achieving over 81% and 2630 mmol h^−1^ g^−1^ for formaldoxime production. This platform upcycles pollutants into valuable chemicals, holding the promise to enable sustainable synthesis of more functionally diverse commodities.

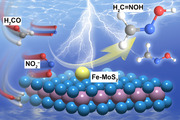

## 1 Introduction

A circular economy via upcycling pollutants with net‐zero emission has become a worldwide goal, evidenced by the initiatives launched by 195 nations to achieve a sustainable society [1]. Compared to the conventional recycling methods, upgrading C‐wastes and N‐toxins into C,N‐feedstocks represents a novel approach toward achieving resource neutrality [2, 3, 4, 5, 6, 7, 8, 9, 10]. In the previous decade, notable advancements have been realized in the fields of electrochemical CO_2_ reduction reaction (CO_2_RR) as well as nitrate and nitrite reduction reactions (NO_3_RR and NO_2_RR) [11, 12, 13, 14, 15, 16, 17, 18, 19, 20]. Specifically, a prominent breakthrough is the substitution of state‐of‐the‐art Pt‐based catalysts with non‐precious metal (NPM) counterparts featuring Cu, Co, Ni, and Fe with enhanced electrocatalytic performance [12, 21, 22, 23, 24, 25, 26]. Cu nanomaterials can generate C_1+_ products such as C_2_H_4_ and propanol from CO_2_ through on‐surface C–C bond formation, while Ni, Co, and Fe can form NH_3_ and other partially reduced N‐products from NO_3_^−^ exclusively [27, 28, 29, 30, 31, 32]. Hence, utilizing applied potentials or currents powered by renewable energy sources under benign conditions to facilitate the upgrading process offers a green approach for independent resourcification of C‐waste into energy‐rich fuels and N‐toxin into fertilizers [33, 34].

Since C‐only and N‐only reductions have gained promising traction in recent decades, electrocatalysis is envisioned to form more structurally complex and functionally diverse C,N‐products that are highly desired in the fine chemicals and textile industries [35]. Utilizing CO_2_ as a C source, urea can be generated in aqueous solution with N sources such as NO_2_^–^ (FE: 43%), NO_3_^–^ (FE: 53%), and N_2_ (FE: 9%) [36, 37, 38]. CO can also be converted into urea in the presence of NH_3_ on Pt with an optimal selectivity of approximately 70% [39]. While urea is the most studied co‐reduction C,N‐product, fewer examples have been reported on the generation of other C,N‐products such as methylamine (FE: 13%), formamide (FE: 46%), acetamide (FE: 38%), leucine (FE: 32%), and alanine (FE: 61%) using Co, Cu, or Fe‐electrocatalysts [40, 41, 42, 43, 44]. Although the C‐/N‐atom efficiencies could be high, the scope and FE for desired products still have substantial room for improvement.

Previous attempts mostly focus on products with C–N single bonds; however, relatively fewer efforts have been attempted to yield products with C═N double bonds, such as oxime. Formaldoxime (H_2_C═NOH) is an organic compound characterized by the C═N bonds that belongs to the class of imines, which is widely used in the conversion process of aryl diazonium salts to aryl aldehydes in synthetic chemistry, fuel, and medicine industries [45]. Recently, a cobalt β‐tetraaminophthalocyanine/carbon nanotube (CoPc‐NH_2_/CNT) hybrid material has been reported as an electrocatalyst to form H_2_C═NOH (FE: 6%) from CO_2_ and NO_3_^–^ [40]. Zou et al. showed that a Fe catalyst achieves a high yield of near 99% with an FE for benzaldoxime of 12% after 12 h of C═N coupling between aldehydes and NO_x_, which verified the high activity of the Fe‐based catalyst for trapping NO_x_ species to promote further reduction and C═N coupling [46]. These reports present novel insights into the C–N coupling reaction and offer guidance on metal choices for oxime synthesis. However, the low selectivity and efficiency of oxime electrosynthesis necessitate urgent breakthroughs. Additionally, the existing work warrants deeper exploration of whether catalyst structure contributes to C═N formation in reaction efficiency and selectivity. We aim to enhance the selectivity of C═N coupling and improve the understanding of the electrosynthesis process by focusing on catalyst structural design and dopant selection.

**FIGURE 1:**
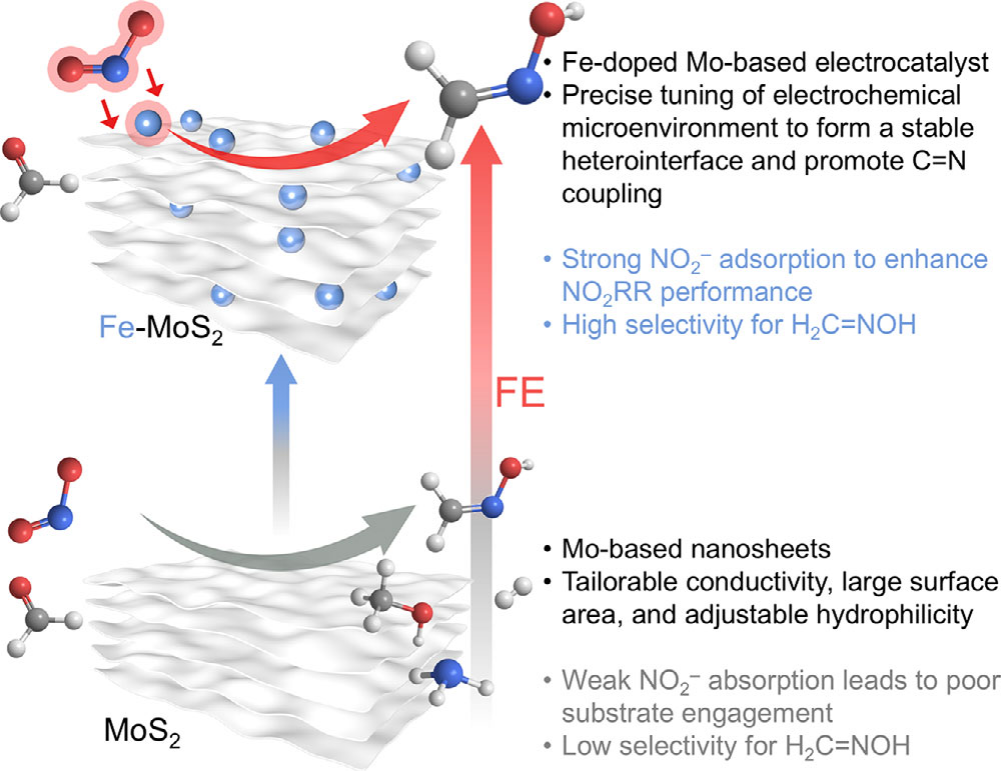
Schematic diagrams of H_2_C═NOH electrosynthesis using Fe‐MoS_2_ versus MoS_2_ as catalysts.

This work focuses on designing catalysts and selecting appropriate N sources to promote the desired electrocatalytic C═N bond formation. N‐intermediates generated from NO_3_RR and NO_2_RR are pivotal factors in the oxime synthesis pathway [47, 48]. In particular, NO_2_RR is favored over NO_3_RR due to the sluggish reduction kinetics involved in the conversion from NO_3_^–^ to NO_2_^–^ [49, 50]. Therefore, NO_2_^–^ is chosen as the N source to conduct the H_2_C═NOH electrosynthesis. The catalyst design is motivated by the structures and properties of NO_3_^–^ reductases that feature Mo and Fe heme active sites in nature [51]. Inspired by the active site structure of nitrate reductases, we rationally designed biomimetic catalysts with dual‐metal active sites to improve the C═N bond formation efficiency. Herein, we report an electrochemical domino process to convert NO_2_^–^ and formaldehyde (H_2_CO) into H_2_C═NOH with Fe‐MoS_2_ as catalysts (Figure 1). The strategy of Fe doping on MoS_2_ surface is envisioned to optimize the absorption of reactants and production of N‐intermediates by regulating the catalyst microenvironment, thus enhancing the yield and selectivity of C═N formation [52].

## 2 Results and Discussion

**FIGURE 2:**
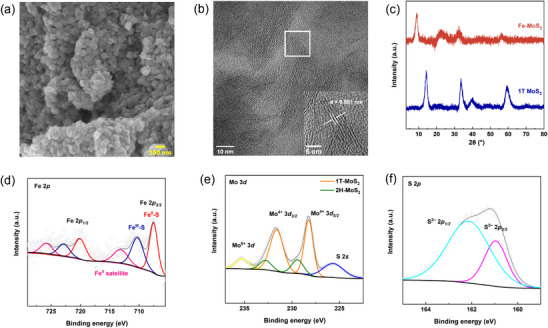
(a) SEM image of Fe‐MoS_2_. (b) Magnified STEM image of Fe‐MoS_2_. An inset highlighting the lattice fringes. (c) PXRD spectra of Fe‐MoS_2_ and MoS_2_. (d) High‐resolution Fe 2p spectrum of Fe‐MoS_2_. (e) High‐resolution Mo 3d XPS spectrum of Fe‐MoS_2_. (f) High‐resolution S 2p spectrum of Fe‐MoS_2_.

Inspired by Mo and Fe metalloreductases, dual‐metal Fe‐MoS_2_ nanosheets are designed for electrocatalytic C═N bond formation and synthesized using a one‐pot hydrothermal method. Scanning electron microscopy (SEM), energy‐dispersive x‐ray spectroscopy (EDS), scanning transmission electron microscopy (STEM), and powder X‐ray diffraction (PXRD) are performed to characterize the morphology and composition of Fe‐MoS_2_ nanosheets coated on carbon cloth. The SEM image in Figure 2a shows that Fe‐MoS_2_ displays a layered structure with thin nanosheets (thickness within nanoscale 1–100 nm) stacking together. This observation is further verified using atomic force microscope (AFM), showing that the synthesized Fe‐MoS_2_ has a thickness of approximately 5 nm (Figure S1). EDS elemental mapping in Figure S1 reveals that Fe, Mo, and S are uniformly distributed on Fe‐MoS_2_ nanosheets (Figure S2). SEM and EDS results verified the presence of Fe atoms doped on the layered nanosheets in Fe‐MoS_2_. EDS mapping and ICP‐MS suggest that the atomic ratio between Fe and Mo is nearly 0.075:1 (Table S1). To explore the effect of metal doping on the interlayer distance of pristine MoS_2_, STEM is first applied to obtain the lattice fringes of pristine MoS_2_ and Fe‐MoS_2_. STEM images in Figure 2b and Figure S3 show that the interlayer distances of pristine MoS_2_ and Fe‐MoS_2_ are 0.638 nm and 0.880 nm, respectively. The 0.242 nm increase is ascribed to the Fe dopant on the MoS_2_ nanosheets. The interlayer expansion phenomenon is further validated using PXRD by deducing the interlayer distance from the (002) peak. PXRD results show a similar interlayer distance expansion of 0.247 nm (Figure 2c and Table S2). According to the XRD results and STEM images, the expansion of the (002) interlayer spacing of Fe‐MoS_2_ is approximately 0.24 nm, which is nearly twice the atomic radius of Fe (around 0.12 nm), indicating the intercalation of Fe atoms between MoS_2_ layers, reminiscent of a case reported in literature [53]. When the Fe:Mo ratio in Fe‐MoS_2_ is changed from 0.025:1 to 0.125:1, the (002) peak does not have an obvious shift, indicating that the effect of the doping percentage of Fe in Fe‐MoS_2_ has no effect on the basal spacing expansion (Figure S4). Since the interlayer distances deduced from PXRD are consistent with those determined using STEM, we further use PXRD to explore the interlayer expansion phenomenon of MoS_2_ nanosheets upon surface doping with metals other than Fe (Figure S5). PXRD results show that a similar interlayer expansion is observed upon introducing Ni, Mg, Zn, Cu, Al, Co, and Ru to MoS_2_ nanosheets. The STEM and PXRD results corroborate that after introducing metal cations into interlayers of pristine MoS_2_, the interlayer spacings will be expanded with metal dopants as spacers.

X‐ray photoelectron spectroscopy (XPS) is performed to further elucidate the chemical composition and oxidation state of the metals in the Fe‐MoS_2_ nanosheets. Mo, S, and Fe can be identified in the XPS spectra, consistent with the EDS results. The two predominant peaks with binding energies at around 708 and 720 eV are assigned to Fe 2p_3/2_ and Fe 2p_1/2_, respectively. The Fe 2p spectra are fitted well with peaks located at 707.4 eV (Fe(II)–S), 711.0 eV (Fe(III)–S), and 713.1 eV (satellite of Fe(II)–S), which shows the co‐existence of Fe(II) and Fe(III), similar to two examples published in the literature [54, 55]. As shown in Figure 2e, the splitting pattern observed in the Mo 3d region suggests the co‐existence of 1T phase MoS_2_ and 2H phase MoS_2_, reminiscent of literature precedence [56]. Two major peaks at 228.3 eV and 231.6 eV in the Mo 3d_5/2_ and 3d_3/2_ regions correspond to the 1T phase MoS_2_, indicating the presence of Mo^4+^ bound to sulfur. In addition, another two minor peaks at 229.4 and 233.4 eV are attributed to the 2H phase MoS_2_. The peak at 236.08 eV is attributed to Mo^6+^, likely generated via atmospheric oxidation of Mo^4+^. Lastly, two characteristic peaks at 160.9 and 162.1 eV are attributed to the binding energies of S 2p_3/2_ and 2p_1/2_, indicating the presence of S^2–^, matching previously reported cases (Figure 2f). To further investigate the morphology of Fe‐MoS_2_, Raman spectroscopy is employed to probe the phase of MoS_2_ in Fe‐MoS_2_ (Figure S6). Compared to as‐prepared 1T phase MoS_2_, the spectrum of Fe‐MoS_2_ exhibits three characteristic peaks of 146, 281, and 335 cm^−1^ as *J*_1_, *J*_2_, and *J*_3_, which is consistent with the phonon modes of 1T MoS_2_ [56]. In contrast, the Raman shifts (374 and 402 cm^−1^, respectively) that are associated with the phonon modes in the 2H phase MoS_2_ are detectable with low intensity in the Fe‐MoS_2_ sample [57, 58]. These observations support the results of XPS, verifying the dominant presence of the 1T phase of MoS_2_ in Fe‐MoS_2_. Taken together, these results indicate the successful synthesis of Fe‐MoS_2_ nanosheets with Fe^2+^ doped onto S^2–^ surface sites of 1T phase MoS_2_ and 2H phase MoS_2_.

X‐ray absorption spectroscopy (XAS) further verifies the presence of Fe–S in Figure S7. The Fe K‐edge XANES spectra (Figure S7a) suggest that the oxidation state of Fe in Fe‐MoS_2_ is between FeS and Fe_2_O_3_, supporting the co‐existence of Fe(II) and Fe(III) observed in XPS. The corresponding Fourier‐transformed extended x‐ray absorption fine structure (FT‐EXAFS) spectroscopy (Figure S7b) reveals that the first coordination shell in both samples is dominated by Fe–S scattering. No significant Fe–Fe coordination signal is detected, suggesting the absence of metallic Fe clusters or nanoparticles. These results are further supported by the wavelet‐transform (WT) data of EXAFS data at the Fe K‐edge (Figure S8). Consequently, the Fe atom is intercalated between the nanosheets in Fe‐MoS_2_, connecting neighboring MoS_2_ layers via Fe–S bonds.

**FIGURE 3:**
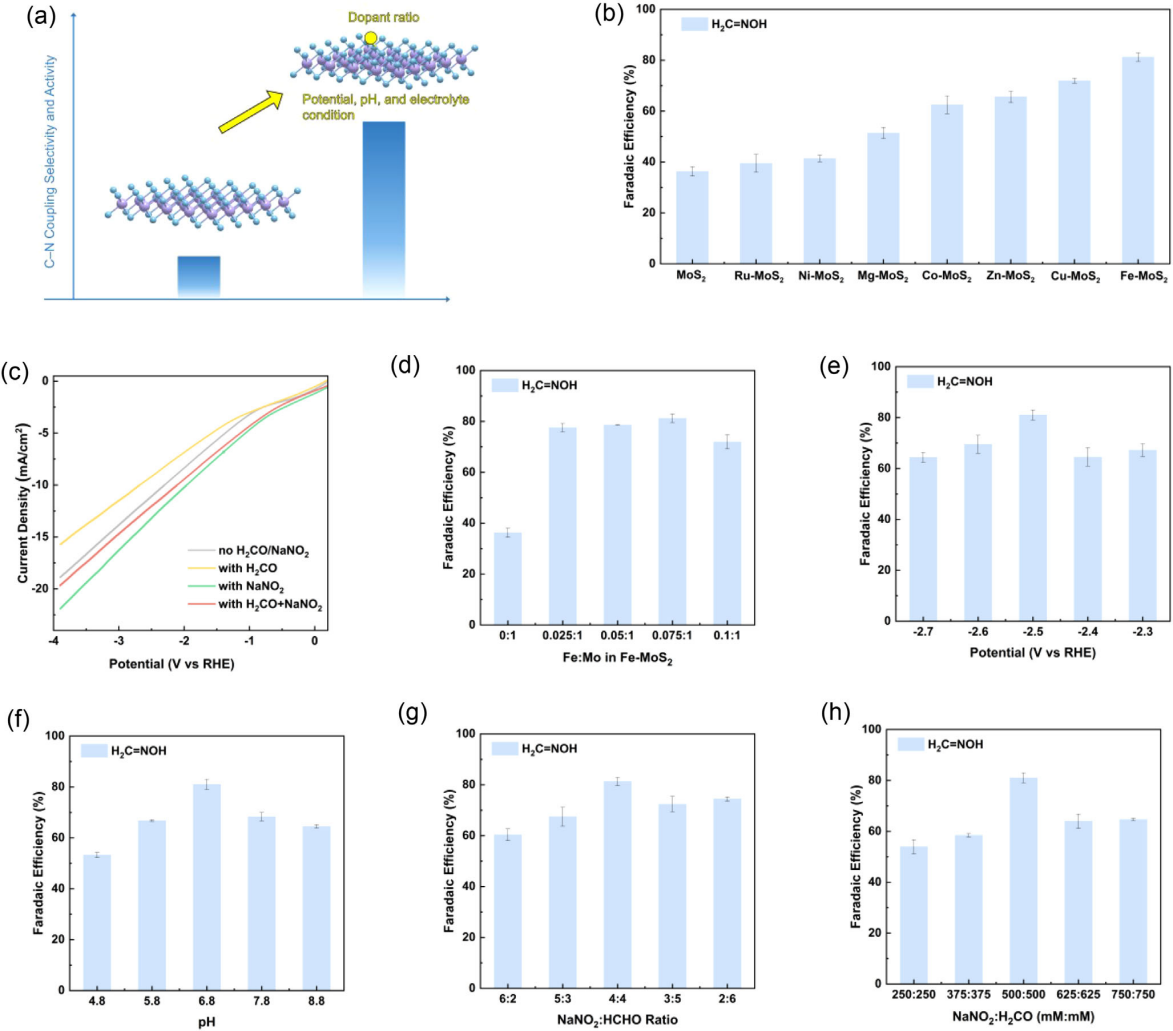
(a) Selectivity and activity comparison between MoS_2_ and Fe‐MoS_2_ conducting C–N coupling reaction. (b) FE for H_2_C═NOH using MoS_2_ and M‐MoS_2_ with 0.5 M NaNO_2_ and 0.5 M H_2_CO in 0.1 M PBS (M═Ru, Ni, Mg, Co, Zn, Cu, and Fe).(c) LSV curves of Fe‐MoS_2_ in four solutions: 0.1 M PBS (gray) containing 0.5 M NO_2_^−^ and 0.5 M H_2_CO (red), 0.5 M NO_2_^−^ only (green), and 0.5 M H_2_CO only (yellow). (d) Effect of Fe:Mo ratio on the FE for H_2_C═NOH on Fe‐MoS_2_. (e) Potential‐dependent FE for H_2_C═NOH on Fe‐MoS_2_. (f) FE for H_2_C═NOH using Fe‐MoS_2_ with a pH of electrolytes increasing from 4.8 to 8.8. (g) FE for H_2_C═NOH using Fe‐MoS_2_ with NaNO_2_:H_2_CO ratio changing from 6:2 to 2:6. (h) FE for H_2_C═NOH using Fe‐MoS_2_ with a concentration of NaNO_2_ or H_2_CO changing from 250 mM to 750 mM.

Electrochemical C–N coupling is first investigated in divided H‐cells using a three‐electrode setup. Electrolysis is conducted to convert NO_2_^−^ and H_2_CO into H_2_C═NOH using carbon cloths coated with M‐MoS_2_ (M═Fe, Cu, Zn, Co, Mg, Ni, and Ru) and MoS_2_ as working electrodes and 0.1 M PBS (pH 6.8) sparged with Ar containing 0.5 M NaNO_2_ and 0.5 M H_2_CO as the electrolyte solution (Figure 3a). After performing electrolysis at –1.9 V versus RHE for 1 h, the gaseous products are quantified by gas chromatography (GC), and soluble products are quantified by nuclear magnetic resonance (NMR) and UV‐vis spectroscopy, respectively. Resonances at 7.10 ppm are consistent with the H_2_C═NOH standard, indicating that the desired product with a newly formed C═N bond is generated (Figure S9). We first explore the dopant identity on MoS_2_ nanosheets for H_2_C═NOH electrosynthesis from NaNO_2_ and H_2_CO (Figure 3b and Figure S10). The dopants of interest are screened based on their capability in reduction reaction and nitrate adsorption from previous reports [59]. In Figure 3b, Co‐MoS_2_, Zn‐MoS_2_, Cu‐MoS_2_, and Fe‐MoS_2_ demonstrate superior catalytic efficiency compared to Mg‐MoS_2_, which is consistent with previous reports highlighting the key roles of transition metals in catalyzing NO_3_^–^/NO_2_^–^ reduction reaction (NO_3_RR/NO_2_RR). Thoroughly speaking, M‐MoS_2_ (M═Fe, Cu, Zn, Co, Mg, Ni, and Ru) with dopants establishes a significant enhancement in C–N coupling compared to pristine MoS_2_. These findings are supported by previous research indicating that Mo‐based and Fe‐based NO_x_ reduction enzymes and electrocatalysts exhibit superior performance in NO_x_ reduction reaction in both natural enzymatic processes and electrochemistry [60, 61]. Fe as the most effective dopant can improve the FE for H_2_C═NOH to 81.2% and a selectivity of 78.6% in organonitrogenous products, which represents the highest reported value to date.

The C–N coupling performance of Fe‐MoS_2_ and MoS_2_ is next evaluated using linear scanning voltammetry (LSV). Compared with MoS_2_, the Fe‐MoS_2_ showed a higher current density and a more positive onset potential in a 0.1 M PBS solution containing HCHO and NaNO_2_, suggesting that Fe‐MoS_2_ exhibits a higher activity for the C–N coupling process (Figure S11). To further obtain insights into the catalytic activity of Fe‐MoS_2_ for the conversion of NO_2_^–^ and H_2_CO into H_2_C═NOH, LSV curves are recorded in four solutions: 0.1 M PBS (gray) containing 0.5 M NO_2_^–^ and 0.5 M H_2_CO (red), 0.5 M NO_2_^–^ only (green), and 0.5 M H_2_CO only (yellow) using Fe‐MoS_2_ as catalysts. As shown in Figure 3c, a more positive onset potential and a higher current density are observed in the presence of NO_2_^–^ upon comparing the LSV curves in 0.1 M PBS solutions with NO_2_^–^ only (green) and H_2_CO only (yellow). This difference suggests that the NO_2_^–^ reduction reaction (NO_2_RR) is more thermodynamically favored. Notably, when compared to the 0.1 M PBS solution alone (gray), the onset potential shifts significantly in the positive direction and the current density increases in the presence of NO_2_^–^ (green), which is indicative of the high performance of NO_2_RR. Similar observations are made upon the introduction of both NO_2_^–^ and HCHO (red) into 0.1 M PBS solutions (gray), with an improvement in the onset potential and an increase in the current density, indicating that the C═N coupling process is energetically more favorable than the hydrogen evolution reaction (HER).

To further verify that the observed change in current density originates from the Fe dopant, a comparative evaluation of H_2_C═NOH electrosynthesis performance is conducted on MoS_2_ with different Fe doping levels. Figure 3d and Figure S12 show the electrosynthesis of H_2_C═NOH from H_2_CO and NO_2_^–^ using MoS_2_ and Fe‐MoS_2_ with varying Fe:Mo ratios. MoS_2_ is found to display a Faradaic efficiency (FE) for H_2_C═NOH of 36%, which is already the highest reported in literature (previous FE record: 6%). Upon Fe doping, Fe‐MoS_2_ generates H_2_C═NOH with a FE close to 80%, which is more than twice that of MoS_2_. This recording‐breaking observation underscores the crucial influence of regulating the microenvironment of the MoS_2_ catalytic surface in enhancing the H_2_C═NOH electrosynthesis process. By adjusting the ratio of Fe:Mo of Fe‐MoS_2_, we find that a Fe:Mo ratio of 0.075:1 yields the maximal FE for H_2_C═NOH of 81.2% and the maximal yield rate (YR) for H_2_C═NOH reaches 963 mmol h^−1^ g^−1^ at –2.5 V versus RHE. Therefore, the optimal Fe:Mo ratio of 0.075:1 with record‐breaking FE and YR is used in subsequent studies on the co‐electrolysis of NO_2–_ and H_2_CO to generate H_2_C═NOH.

We optimize the C–N coupling performance of Fe‐MoS_2_ by exploring the influence of applied potential, solution pH, substrate concentration, and dopant identity. First, by tuning the applied potential, a volcanic relationship between selectivity and activity for H_2_C═NOH and applied potential is observed from –2.7 to –2.3 V versus RHE (Figure 3e and Figure S13). The highest FE for H_2_C═NOH is obtained at –2.5 V versus RHE, likely too low of a driving force results in poor NO_2_RR while too high of an overpotential renders the competitive HER to become the dominant reaction. Second, we tailor the interfacial conditions by changing the pH of the electrolyte solutions. Both acidic and alkaline conditions have negative impacts on the C–N coupling performance, whereas the optimal selectivity and activity are achieved at pH 6.8 (Figure 3f and Figure S14). This observation likely stems from HER overtaking electrochemical C–N coupling as the dominant reaction in acidic conditions, while formaldoxime is unstable in alkaline conditions. Importantly, neutral pH provides a suitable coverage of surface H* to promote the partial reduction of NO_2_^–^ into N‐intermediates poised for C–N coupling. Third, while holding the total concentrations of N source and C source to be 1 M, we adjust the ratio between N source and C source. In Figure 3g and Figure S15, when the concentration of N source exceeds that of C source or vice versa, the FE and YR for H_2_C═NOH decrease. The results show that peak C–N coupling efficiency is attained at a 1:1 molar ratio of NO_2_^−^ and H_2_CO, resulting in a well‐balanced surface concentration of N‐species and C‐species for C–N bond formation. Fourth, the overall concentration of NO_2_^−^ and H_2_CO is further modified (Figure 3h and Figure S16). When the total concentration of NO_2_^–^ and H_2_CO increases to 1 M, the FE and YR for H_2_C═NOH increase. However, a further increase in the total concentration of NO_2_^–^ and H_2_CO leads to a decrease in overall efficiency. This phenomenon is likely due to HER becoming the dominant reaction when the total concentration of the C and N sources is too low, while excess NO_2_^–^ and H_2_CO diminishes surface H* coverage that is needed for co‐reduction. The side products of H_2_CO reduction reaction and NO_2_RR are also investigated. Methanol, NH_4_^+^, and NH_2_OH are detected in the electrolyte after 1 h electrolysis (Figures S17–S26, see Supporting Information Note 14). To further investigate the long‐term operational durability of Fe‐MoS_2_ for H_2_C═NOH electrosynthesis, a 10‐cycle continuous electrolysis test is performed at –2.5 V versus RHE (Figure S27). In Figure S27a, the FE for H_2_C═NOH remains stable without notable decline. Additionally, the catalytic current density for oxime electrosynthesis remains unchanged after 10 successive cycles (Figure S27b). These results collectively demonstrate the high robustness of Fe‐MoS_2_ for H_2_C═NOH electrosynthesis. Therefore, the optimal microenvironment condition for H_2_C═NOH electrosynthesis is systematically found to be 0.1 M phosphate‐buffered saline (PBS) at pH 6.8 with 0.5 M H_2_CO and 0.5 M NaNO_2_ at –2.5 V versus RHE.

Control experiments are systematically carried out to address three underlying questions: (1) what the source of protons on the carbon of H_2_C═NOH is, (2) which N species are accountable for the C–N coupling reaction, and (3) whether H_2_ produced from competing hydrogen evolution reaction (HER) can facilitate C═N bond formation. Regarding the proton origin on the carbon of H_2_C═NOH, cross isotopic labeling experiments are employed (Figure S13; entries 1, 2, 3, and 4 in Table 1). The resonances of H_2_C═NOH are only detected when the substrate is H_2_CO in ^1^H NMR, while the resonances of D_2_C═NOH are only detected when the substrate is D_2_CO in ^2^D NMR. The same observations are recorded regardless of whether H_2_O or D_2_O is used as the solvent. Since scrambling is not observed, these results verify that the protons on the carbon of H_2_C═NOH are derived from the H_2_CO reactant rather than water and that the protons are non‐exchangeable with water. To address the second question, N‐species like NO, NH_2_OH, and NH_3_ that can be produced during NO_2_RR pathway are used to replace NO_2_^–^ as the reactant to conduct H_2_C═NOH electrosynthesis (Figures S28–S32; entries 5, 6, 7, and 8 in Table 1). When the N sources are NO and NH_2_OH, H_2_C═NOH is detected after 1 h of electrolysis, suggesting a tandem pathway where NO_2_^–^ is first reduced into NO, which is subsequently reduced into NH_2_OH, which then condenses with H_2_CO to form H_2_C═NOH. To address the final question, the reactor is saturated with H_2_ without applied potential nor current (Figure S33; entry 9 in Table 1). After 1 h, H_2_C═NOH is not detected. Hence, the NO_2_RR process is driven by the applied potential and current rather than the H_2_ generated from HER. Overall, these control experiments elucidate the proton‐coupled electron transfer mechanism at the electrochemical interface and underscore the importance of facilitating NO_2_RR using applied potential and current during H_2_C═NOH electrosynthesis.

**TABLE 1:** List of control experiments.

Entry	N source	C source	Water	Electricity	C–N products
1	NO_2_^−^	H_2_CO	H_2_O	Yes	H_2_C═NOH
2	NO_2_^−^	H_2_CO	D_2_O	Yes	H_2_C═NOH
3	NO_2_^−^	D_2_CO	H_2_O	Yes	D_2_C═NOH
4	NO_2_^−^	D_2_CO	D_2_O	Yes	D_2_C═NOH
5	NO	H_2_CO	H_2_O	Yes	H_2_C═NOH
6	NH_2_OH	H_2_CO	H_2_O	Yes	H_2_C═NOH
7	NH_2_OH	H_2_CO	H_2_O	No	H_2_C═NOH
8	NH_4_^+^	H_2_CO	H_2_O	Yes	No
9	NO_2_^−^	H_2_CO	H_2_O	No (H_2_ condition)	No

**FIGURE 4:**
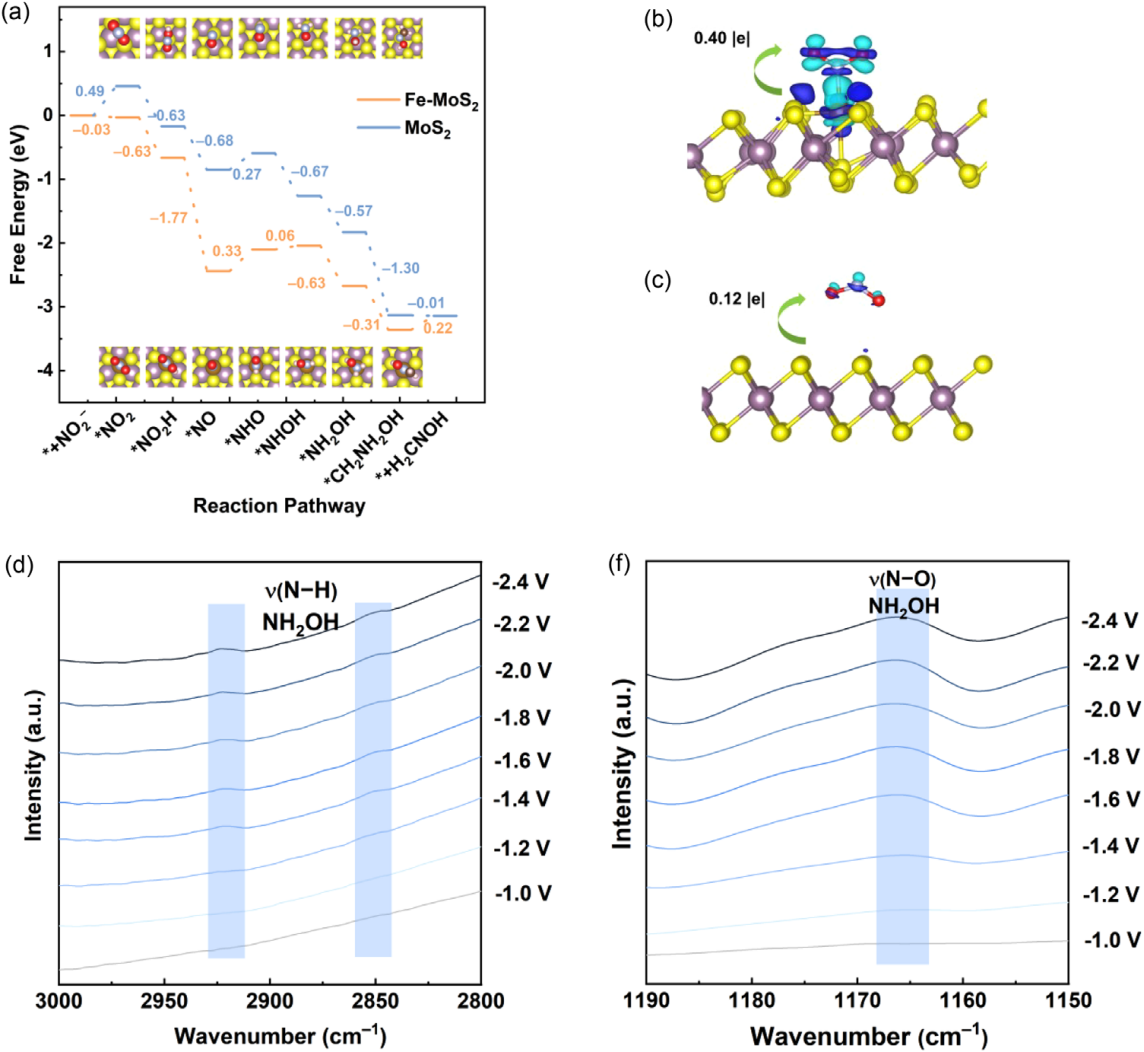
(a) Free energy diagram for formaldoxime electrosynthesis on Fe‐MoS_2_ and MoS_2_. Charge difference of (b) Fe‐MoS_2_ and (c) MoS_2_ with adsorbed NO_2_^–^. ATR‐FTIR spectra for the electrocatalytic C═N coupling from H_2_CO and NO_2_^−^ over the Fe‐MoS_2_ (d) from 2800 to 3000 cm^−1^ and (e) from 1500 to 1190 cm^−1^.

In our density functional theory (DFT) simulations, we thoroughly explore the intricate binding properties and free energy landscapes of Fe‐MoS_2_ and MoS_2_ catalysts. Our calculations unveil that MoS_2_ exhibits a remarkable propensity for binding Fe atoms on the surface of MoS_2_, thus forming a stable Fe‐MoS_2_ structure. For MoS_2_, the initial NO_2_^–^ binding step is the potential‐determining step (PDS) with an energy penalty of 0.49 eV, as illustrated in Figure 4a. Upon introducing Fe into MoS_2_ to form Fe‐MoS_2_, a substantial enhancement in NO_2_^−^ binding is observed with an adsorption energy of –0.03 eV. Therefore, the Fe‐MoS_2_ composite exhibits a superior binding affinity toward NO_2_^–^ compared to MoS_2_. This boosted adsorption capacity of Fe‐MoS_2_ prompted the cleavage of the N–O bond in the *NO_2_H intermediate. Subsequently, protonation of the adsorbed nitric oxide (*NO) becomes the PDS on Fe‐MoS_2_ (0.33 eV), which is less than the 0.49 eV required for the PDS of the MoS_2_ system. To provide further insights into the binding behavior, Bader charge analysis is conducted (Figure 4b,c). This analysis reveals a notable charge transfer of 0.40 |e| from Fe‐MoS_2_ to NO_2_^–^, while the adsorbed MoS_2_ system displays a lower charge transfer of only 0.12 |e|. These findings underscore the pivotal role of the introduction of Fe in enhancing electron transfer at the electrochemical interface, thereby amplifying the adsorption capacity of NO_2_^–^. By integrating the Bader charge analysis results and the free energy diagrams, we gain a comprehensive mechanistic understanding of how the incorporation of Fe improves the adsorption of NO_2_^–^ and promotes the formation of oxime products within the catalyst microenvironment of Fe‐MoS_2_.

To further understand the reaction pathway of H_2_C═NOH electrosynthesis on Fe‐MoS_2_, electrochemical in‐situ attenuated total reflectance Fourier transform infrared (ATR‐FTIR) spectroscopy is employed to identify key intermediates (Figure 4d,e and Figure S34). As the applied potential increases, two absorption bands emerge at 2850 and 2923 cm^−1^, corresponding to the N−H stretching vibrations of *NH_2_OH [46]. A new band simultaneously appears at 1165 cm^−1^, which is assigned to the N–O stretch of *NH_2_OH [62]. Our in‐situ IR analysis indicates the formation of key intermediate NH_2_OH during the C═N coupling reaction on Fe‐MoS_2_, which is also consistent with our control experiments and DFT calculation results (see Supporting Information Note 20).

**FIGURE 5:**
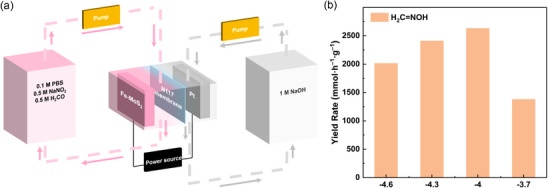
(a) Schematic diagram of a membrane electrode assembly (MEA) fluidic device for flow‐based electrocatalytic C,N‐upcycling. (b) Potential‐dependent YR for H_2_C═NOH on Fe‐MoS_2_ in the flow‐cell prototype for continuous co‐electrolysis for 1 h.

To assess the industrial feasibility of using Fe‐MoS_2_ to mediate electrocatalytic C–N coupling for large‐scale production of H_2_C═NOH, scale‐up electrolysis is performed in a 50‐mL flow‐cell prototype (Figure 5a). After continuous operation for 1 h in the membrane electrode assembly (MEA), the YR for H_2_C═NOH is enhanced from 963 mmol h^−1^ g^−1^ (static three‐electrode H‐cell setup) to beyond 1300 mmol h^−1^ g^−1^ (two‐electrode flow cell reactor) (Figure 5b). The applied potential across the two electrodes is further optimized. Upon increasing the driving force from –3.7 to –4.0 V, the YR drastically increases to a record‐high 2630 mmol h^−1^ g^−1^. However, a decrease in YR is noted when the overpotential increases further, likely due to competing reactions such as HER and methanol reduction overtaking as the dominant reactions. Acetaldoxime, 2‐propanone oxime, and cyclohexanone oxime are next targeted as products due to their importance in pharmaceutical industries. Using our flow electrolyzer, we achieve high YR of 965, 758, and 825 mmol h^−1^ g^−1^ for acetaldoxime, 2‐propanone oxime, and cyclohexanone oxime, respectively (Figure S35). Finally, we choose cyclohexanone oxime to verify the industrial applicability of our electrocatalytic C–N coupling on Fe‐MoS_2_. Through our techno‐economic analysis (TEA) calculation (see Supporting Information Note 21 for TEA section), the electrosynthesis of cyclohexanone oxime from cyclohexnanone oxime is estimated to yield a profit of $1144.2/ton. Taken together, the remarkable production rate of H_2_C═NOH in a MEA demonstrator unit reveals the promising utility of Fe‐MoS_2_ toward enabling scalable C═N coupling and the practical applicability of tandem flow electrocatalysis for C,N‐wastes co‐valorization.

## 3 Conclusion

In summary, MoS_2_ nanosheets with metal dopants are designed and developed to electrosynthesize H_2_C═NOH from NO_2_^−^ and H_2_CO. Our work demonstrates that Fe dopant is optimal to improve the C–N coupling reaction with a Faradaic efficiency (FE) for H_2_C═NOH of 81.2% and a yield rate (YR) for H_2_C═NOH of 963 mmol h^−1^ g^−1^. Control experiments reveal that C–N bond formation relies on the successive reduction of NO_2_^−^ driven by applied potential and current while N‐intermediates play crucial roles during this process. Our DFT computational analysis reveals the major roles of Fe dopants in enhancing the absorption of NO_2_^–^, thus facilitating the electrosynthesis of H_2_C═NOH in water at room temperature under ambient pressure. Our study introduces a strategy to successfully boost the electrosynthesis of C═N containing compounds by regulating the microenvironment of electrocatalysts in a green solvent using renewable electricity. Upon incorporating Fe‐MoS_2_ into a flow electrolyzer, the YR for formaldoxime almost triples to a record‐high 2630 mmol h^−1^ g^−1^. A techno‐economic analysis (TEA) is used to assess the translational value of our strategy, showing a profit of $1144.2/ton of cyclohexanone oxime. This work establishes a new sustainable approach using nonprecious metal‐doped catalyst to simultaneously upgrade C‐ and N‐pollutants and generate value‐added organonitrogenous feedstocks via electrocatalysis powered by clean energy sources, establishing a dual‐purpose route to sustainable manufacturing.

## Conflicts of Interest

The authors declare no conflicts of interest.

## Supporting information




**Supporting File 1**: anie71964‐sup‐0001‐SuppMat.Pdf.

## Data Availability

The data that support the findings of this study are available from the corresponding author upon reasonable request.
