# Supplementary material for: Convergent Valorization via Scalable C═N Bond Electrosynthesis Through Metal‐Dopant Interfacial Engineering in a Flow Electrolyzer
Source: Angew Chem Int Ed Engl. 2026 Mar 30;65(20):e9964729. doi: 10.1002/anie.9964729 (PMC13159428; doi:10.1002/anie.9964729)
Supplement: Supplementary file 1 — Supporting File 1: anie71964‐sup‐0001‐SuppMat.Pdf. [file ANIE-65-e9964729-s001.pdf]

Convergent Valorization via Scalable C=N Bond Electrosynthesis through Metal-Dopant Interfacial Engineering in a Flow Electrolyzer

Ruijie Yi,[a] Jiu Chen, [a] Xiaoyong Mo, [a] Tian Zeng,[b] Jingtao Zhou, [c] Fulai Liu, [c] [d] Shu-Chih Haw,* [e] Yong Chen,* [c] [d] Ruosong Li,*[b] Edmund C. M. Tse*[a]

[a] R. Yi, J. Chen, X. Mo, Dr. S.-C. Haw, Prof. E. C. M. Tse

Department of Chemistry, HKU-CAS Joint Laboratory on New Materials

University of Hong Kong

Hong Kong SAR, China

E-mail: ecmtse@hku.hk

[b] T. Zeng, Prof. R. Li

School of Chemical Engineering, Northwest University, Xi'an, Shaanxi 710069, China

E-mail: ruosongli@nwu.edu.cn

[c] J. Zhou, Dr. F. Liu, Prof. Dr. Y. Chen

Key Laboratory of Photochemical Conversion and Optoelectronic Materials & CAS-HKU Joint Laboratory on New Materials

Technical Institute of Physics and Chemistry, Chinese Academy of Sciences

Beijing 100190, P. R. China

E-mail: chenyong@mail.ipc.ac.cn

[d] J. Zhou, Prof. Dr. Y. Chen

University of Chinese Academy of Sciences

Beijing 100049, P. R. China

[e] Dr. S.-C. Haw

National Synchrotron Radiation Research Center (NSRRC)

101 Hsin-Ann Road, Hsinchu Science Park, Hsinchu 30076, Taiwan

Table of Contents – SI Notes

1.	Experimental Section	3
2.	AFM of Fe-MoS_2	8
3.	EDS Mapping of Fe-MoS_2	9
4.	ICP-MS and EDS of Fe-MoS_2	10
5.	TEM Images of MoS_2	11
6.	XRD of Fe-MoS_2 with Different Fe Concentration	12
7.	XRD Spectra of M-MoS_2 and MoS_2 (M = Fe, Cu, Zn, Co, Mg, Ni, and Ru)	13
8.	Raman Spectra of Fe-MoS_2, 1T MoS_2, and 2H MoS_2	15
9.	XAS Spectra of Fe-MoS_2	16
10.	^{1}H NMR of $H_2C{=}NOH$	20
11.	FE for $H_2C{=}NOH$, CH_3OH, NH_4^+, NH_2OH, and H_2 Using M-MoS_2 (M = Fe, Ni, Mg, Zn, Cu, Co, and Ru) as Catalysts	21
12.	LSV Curves of Fe-MoS_2 (red) and MoS_2	22
13.	YR for $H_2C{=}NOH$ Using Fe-MoS_2 as Electrocatalyst	23
14.	FE for Methanol, NH_4^+, and NH_2OH Using Fe-MoS2 as Electrocatalyst	28
15.	YR for Methanol, NH_4^+, and NH_2OH Using Fe-MoS_2 as Electrocatalyst	33
16.	Stability test of Fe-MoS_2 as Electrocatalyst	39
17.	^{1}H NMR Spectra and ^{2}D NMR Spectra of the Reaction Solutions in Cross Isotopic Labelling Experiments	41
18.	^{1}H NMR Spectra of the Reaction Solutions in reactants control experiments	42
19.	^{1}H NMR Spectra of the Reaction Solutions in H_2 Control without Applied Potential nor Current	44
20.	Entire in-situ IR Spectra of $H_2C{=}NOH$ Electrosynthesis on Fe-MoS_2	45
21.	Scope Extension on Fe-MoS_2 in the Flow-cell	46
22.	Techno-Economic Analysis Calculation	47

SI Note 1. Experimental Section

Materials

All reagents were purchased from commercial sources without further purification unless otherwise stated. Ar (99.995% high purity grade) and N_2 (99.995% high purity grade) were purchased from Linde HKO. For the synthesis of Fe-MoS_2, ammonium tetrathiomolybdate ($(NH_4)_2MoS_4$) from Bide Pharmaceutical and iron(III) nitrate nonahydrate ($Fe(NO_3)_3 \cdot 9H_2O$) from J&K Scientific were used as the precursors of MoS_2 and Fe-doped MoS_2. Commercial carbon cloth (WOS1009) was used as a conductive catalyst support. Water used in all experiments was purified using a Millipore system. Electrochemical measurements were conducted at pH 6.8 in PBS solutions prepared using KCl (J&K Scientific), NaCl (Dieckmann Chemical), Na_2HPO_4 (Dieckmann Chemical), KH_2PO_4 (Dieckmann Chemical). $NaNO_2$ (Acros Organics) and HCHO (37 wt. % in H_2O, Aladdin) were used as the respective N-source and C-source for electrocatalytic formation of formaldoxime.

Preparation of Fe-MoS_2 and M-MoS_2

Fe-doped MoS_2 was synthesized via a one-pot hydrothermal reaction.[1] In brief, 15 mg of $(NH_4)_2MoS_4$ and 1.75 mg of $Fe(NO_3)_3 \cdot 9H_2O$ were dissolved in 12.5 mL DMF. The mixture was further dispersed in an ultrasonic bath for 5 min to achieve a homogeneous brown solution and transferred into a 25 mL Teflon-lined autoclave. Two pieces of 2×3 cm^2 carbon cloths were then submerged into the Teflon liner. The autoclave was sealed and heated to 190 ºC in an oven for 12 hours before cooling down to room temperature. The carbon cloth was then washed three times with water and ethanol to remove the remaining unreacted precursors. Finally, the carbon cloths coated with Fe-MoS_2 samples were dried at 70 ºC in a vacuum oven overnight. The Fe and Mo contents in Fe-MoS_2 samples were controlled by adjusting the feed ratio of $(NH_4)_2MoS_4$ and $Fe(NO_3)_3 \cdot 9H_2O$. An analogous procedure as above was used to prepare M-MoS_2 (M = Cu, Zn, Co, Mg, Ni, and Ru) by using the corresponding metal salt as the dopant.

Preparation of 1T MoS_2

1T MoS_2 was prepared by dissolving 0.36 g of thiourea, 0.18 g of ammonium molybdate tetrahydrate, and 0.075 g oxalic acid in 30 mL deionized water. After transferring into a 50 mL Teflon-lined autoclave, the reactor was heated to 190 ºC for

18 hours. The solid particles were washed 3 times with water and ethanol, followed by drying at 60 °C overnight in a vacuum oven.[2]

Preparation of 2H MoS_2

The 2H MoS_2 was prepared using a one-step hydrothermal reaction.[3] 5 mmol $Na_2MoO_4{\cdot}2H_2O$ and 15 mmol $CS(NH_2)_2$ were added into 20 mL water and transferred to a 50 mL Teflon-lined autoclave. The system was naturally cooled down after heating at 190 °C for 18 hours. The obtained 2H MoS_2 was washed with water and ethanol, followed by drying at 60 °C overnight in a vacuum oven.

Catalyst Characterization

Powder X-ray diffraction (PXRD) data were recorded using a Rigaku MiniFlex600 diffractometer with Cu Kα radiation (λ = 1.5318 Å) for lattice and basal spacing measurements. A LEO 1530 SEM system was used for scanning electron microscopy (SEM) and energy-dispersive X-ray spectroscopy (EDS). EDS data were analyzed using the Aztec software. Scanning transmission electron microscopy (STEM) was performed using a Thermo Scientific Talos F200X STEM to characterize the morphology and crystalline structure of catalysts of interest. Inductively coupled plasma mass spectrometry (ICPMS) was performed on an Agilent Technologies 7700x instrument for elemental analysis. Metal oxidation states of catalysts were probed using X-ray photoelectron spectroscopy (XPS) and X-ray absorption spectroscopy (XAS). XPS was performed on a Thermo Nexsa G2 X-ray photoelectron spectrometer system using monochromatic Al Kα radiation with all binding energies of the spectra corrected to the C1s peak at 284.6 eV. XAS was acquired on the 20A1 beamline at the NSRRC synchrotron facility. Data reduction, data analysis, and EXAFS fitting were performed and analyzed using the Athena and Artemis programs of the Demeter data analysis packages that utilize the FEFF6 program to fit the EXAFS data.[4-5] The energy calibration of the sample was conducted through standard Fe foil, which as a reference was simultaneously measured. A linear function was subtracted from the pre-edge region, then the edge jump was normalized using the Athena software. The $\chi(k)$ data were isolated by subtracting a smooth, third-order polynomial approximating the absorption background of an isolated atom. The k^2-weighted $\chi(k)$ data were Fourier transformed after applying a Hanning window function (Δk = 1.0). For EXAFS modeling, the global amplitude EXAFS (CN, R, σ^2, and ΔE_0) were obtained by nonlinear fitting, with least-squares refinement, of the EXAFS equation to the Fourier-transformed data

in R-space, using the Artemis software. EXAFS of the Fe foil was fitted and the obtained amplitude reduction factor S_0^2 value (0.706) was set in the EXAFS analysis to determine the coordination numbers (CNs) in sample. For Wavelet Transform analysis, the χ(k) exported from Athena was imported into the Hama Fortran Code. The parameters were listed as follows: R range, 1-4 Å; k range, 0-12 $Å^{-1}$; k weight, 2; and Morlet function with κ=10, σ=1 was used as the mother wavelet to provide the overall distribution. Raman spectra of the 1T MoS_2, 2H MoS_2, and Fe-MoS_2 were recorded using a Witec alpha 300 R with a laser wavelength of 532 nm. AFM samples were prepared by drop casting dispersed Fe-MoS_2 nanosheets onto a clean wafer substrate which was pretreated by Piranha solution (98% H_2SO_4: 30% H_2O_2 in a 3:1 ratio). AFM was performed on a Bruker JPK NanoWizard 4 XP system with a REFSPA-75 tip with spring constant 3 N/m under the AC mode. A 1 μm × 1 μm scan area was acquired at a 512 pixel × 512-pixel resolution.

Electrochemical Measurements

Electrochemical studies were conducted in a standard three-electrode system using a CH Instruments 6072E potentiostat. Pt wire, Ag/AgCl electrode (saturated KCl, 0.197 V versus RHE), and carbon cloth coated with catalysts were used as counter electrode, reference electrode, and working electrode, respectively. The freshly prepared electrolyte solution was sparged with Ar for at least 10 minutes before electrochemical studies. All experiments were at least triplicated. The following equation was used to convert all potentials to the RHE scale:

$$V\ (versus\ RHE) = V\ (versus\ Ag/AgCl) + 0.197\ V + 0.0592\ V \times pH \qquad (1)$$

Product Quantification

After 1-hour electrolysis, the liquid products soluble in H_2O were detected by ^1H NMR on a Bruker 500 MHz instrument with water signal suppression (scans = 32 scans, internal standard = 10 mM potassium benzoate). In a typical test, 400 μL of electrolyte solution after electrolysis was added into a NMR tube containing 100 μL of the internal standard solution. The FE of products was calculated by the area ratio of main product or side product versus internal standard (H_2C=NOH (trans H):~7.10 ppm, CH_3OH:~3.35 ppm, benzoate:7.80 ppm).[6]

N-containing products were detected by UV-vis absorption measurements using an Implen NanoPhotometer NP80. NH_4^+ was quantified by the Berthelot reaction to form

indophenol blue by reacting with hypochlorite and salicylate.[7] 5% NaClO solution was prepared by mixing 2:3 (v/v) 11-14% NaClO:Milli-Q water. A basic solution was prepared by mixing 0.775 M trisodium citrate and 0.25 M NaOH. The 5% NaClO solution and the basic solution were mixed in a ratio of 1:4 to prepare an oxidation solution. In a typical test, 1 mL electrolyte solution after each electrolysis run, 4 mL Milli-Q water, 200 μL of 1 M phenol in ethanol, 200 μL of 0.02 M nitroprusside in Milli-Q water, and 600 μL of the oxidation solution were mixed together. The resultant solution was placed subsequently in the dark for 4 hours. Lastly, 100 μL of the reacted solution was used in the UV-Vis quantification process. The absorbance at 630 nm was used to calculate the concentration of NH_4^+ in order to obtain the FE of NH_3 with the use of a standard curve. NH_2OH was quantified by the following method with the use of $K_3Fe(CN)_6$.[8] 0.2 mL of the electrolyte solution, 0.1 mL of 100 mM KCl solution containing 1 mM $K_3Fe(CN)_6$, and 0.3 mL 25% KOH were mixed and reacted for 7 min. The absorbance at 425 nm of the reacted solution was recorded using UV-Vis spectroscopy. The difference in absorbance between the electrolyte solution before and after each electrolysis run was used to compute the concentration of NH_2OH in order to obtain the FE of NH_2OH.

H_2 generated at the cathode was detected by gas chromatography (GC, Agilent 7890A) equipped with a thermal conductivity detector (TCD) with high-purity Ar as the carrier gas. The FE of H_2 was calculated using the area of the H_2 peak, which was converted into gas volume.

Cross Isotopic Labelling Study

The reaction pathway was investigated by cross isotopic labelling experiments. To probe the origin of the protons in the CH_2 group of $H_2C=NOH$, D_2O and D_2CO were used as substitutes for H_2O and H_2CO, respectively. During the electrolysis, four combinations of reaction conditions were tested: (i) H_2CO in H_2O, (ii) H_2CO in D_2O, (iii) D_2CO in H_2O, and (iv) D_2CO in D_2O. The resultant electrolyte solutions after electrolysis were analyzed by both ^1H NMR and ^2D NMR.

Control Study Under H_2 Atmosphere Without Applied Electricity

To evaluate the significance of applied potential and current as well as rule out the possibility of H_2 generated at the cathode serving as the reducing agent, a control experiment without applied potential nor current was conducted. One piece of carbon

cloth (1×1.5 cm^2) coated with Fe-doped MoS_2 was introduced into the electrolyte solution in a round bottom flask under an H_2 atmosphere. The system was subsequently vacuumed and backfilled with H_2 three times before allowing it to react for 1 hour. After reacting for 1 hour under H_2-saturated condition, the products soluble in the electrolyte solution were analyzed by ^{1}H NMR.

Computational Analysis

Calculations were conducted using the Vienna Ab Initio Simulation Package (VASP) software with the implementation of the projector augmented wave (PAW) method.[9-10] To accurately describe electron exchange-correlation effects, the Perdew-Burke-Ernzerhof (PBE) exchange-correlation functional within the generalized gradient approximation (GGA) framework was utilized.[11] A plane-wave cutoff energy of 500 eV was selected for valence electrons. Calculations employed a 3×3×1 mesh of Gamma k-points for both the MoS_2 slab with and without Fe atom in the Brillouin zone. To minimize interlayer interactions, a vacuum layer of 20 Å was integrated into the slab models. The geometrical structures were iteratively optimized until the convergence criteria for forces (below 0.05 eV/Å) and energies (1.0^{-5} eV) were met. Bader charge analysis was conducted alongside free energy calculations to provide complementary information on the electronic properties of the system.[12] Free energy was calculated as follows:

$$\Delta G = \Delta E + \Delta ZPE - T\Delta S$$

where ΔE, ΔZPE, and ΔS were the difference in electronic energy, zero-point vibrational energy, and entropy, respectively, at 298.15 K.

Scale-up Electrolysis in a Flow Reactor

Scale-up electrochemical C–N coupling was conducted in a continuous-flow configuration using a two-compartment electrolyzer (Changsha Sipulin Co., Ltd., China). 0.1 M PBS, 0.5 M $NaNO_2$, and 0.5 M H_2CO was used as the catholyte, while 1 M NaOH was used as the anolyte. The two compartments of the membrane electrode assembly (MEA) were separated by a Nafion-117 membrane. A piece of carbon cloth coated with Fe-MoS_2 and a Pt foil were used as cathode (1 cm^2) and anode (1 cm^2), respectively. The catholytes and anolytes of the flow cell were circulated using two identical peristaltic pumps (KSP-F01A, Kamoer) for 1 hour (flow rate = 1.4 mL/min).

SI Note 2. AFM of Fe-MoS_2

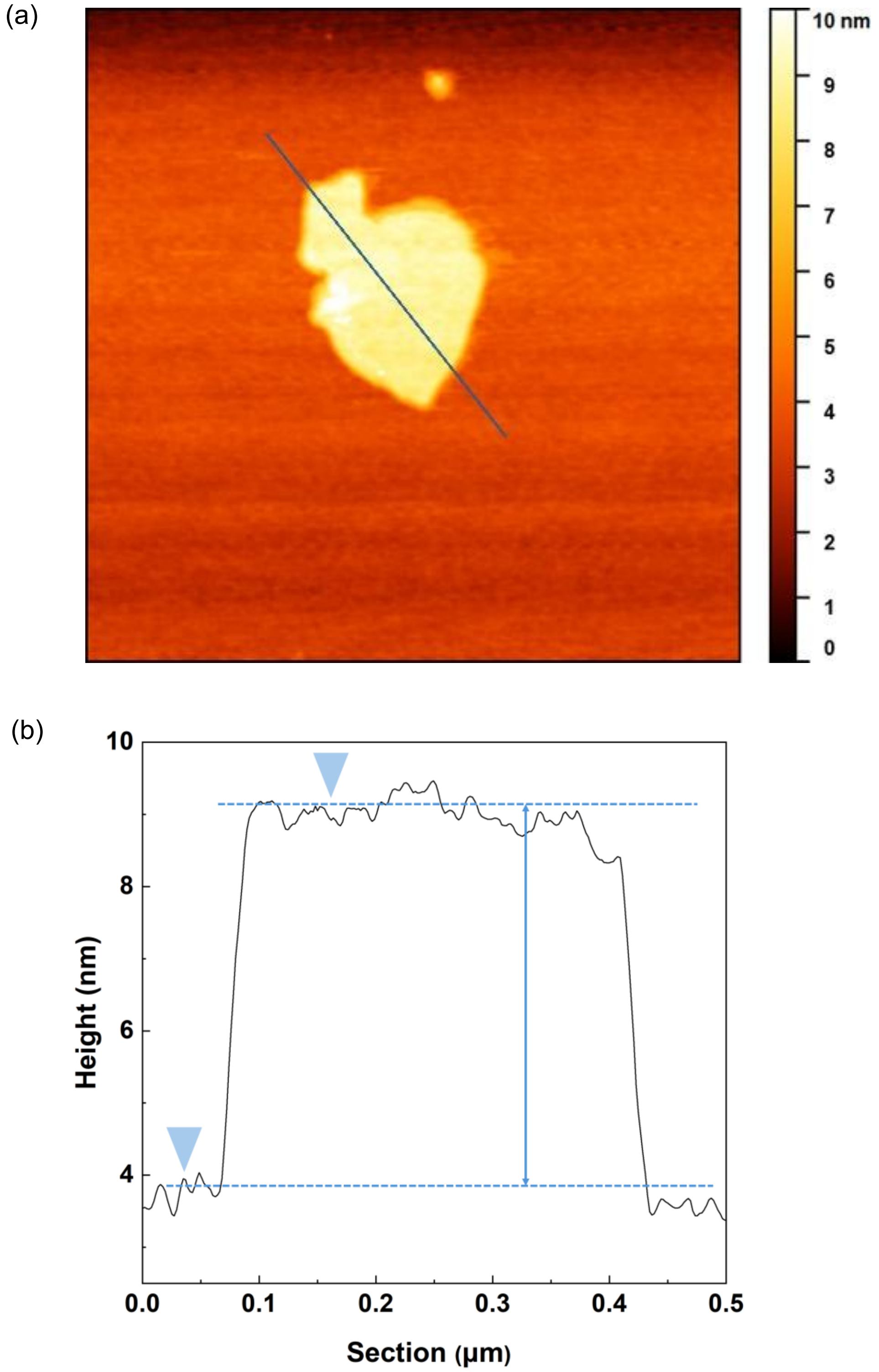

Figure S1. (a) AFM image of Fe-MoS_2. (b) Thickness measurements of Fe-MoS_2 (Z scale) of selected areas.

SI Note 3. EDS Mapping of Fe-MoS_2

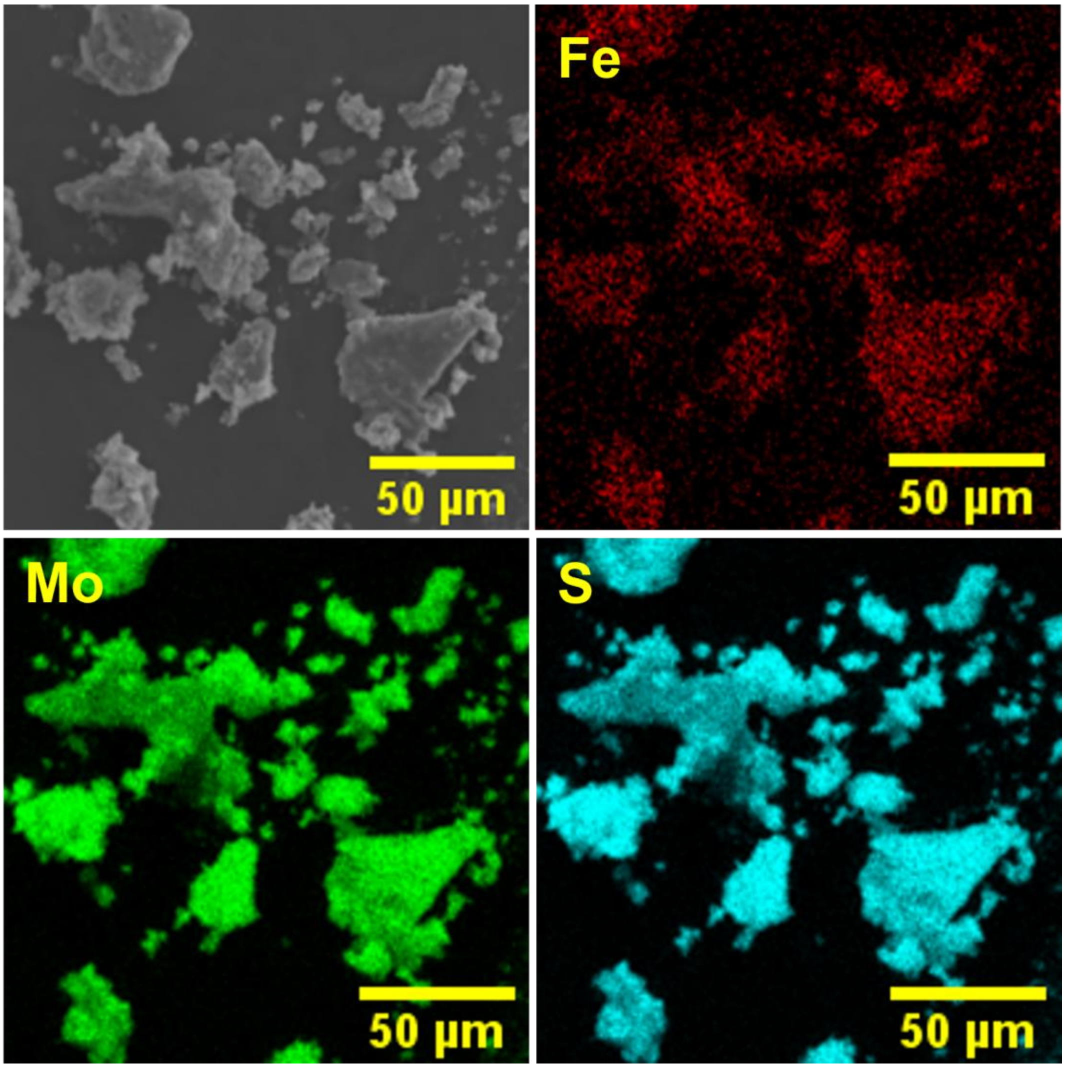

Figure S2. EDS elemental mapping of Fe-MoS_2.

SI Note 4. ICP-MS and EDS of Fe-MoS_2

Table S1. Fe:Mo ratio obtained using ICP-MS and EDS.

Method	Fe:Mo
ICP-MS	0.077:1
EDS	0.076:1

SI Note 5. TEM Images of MoS_2

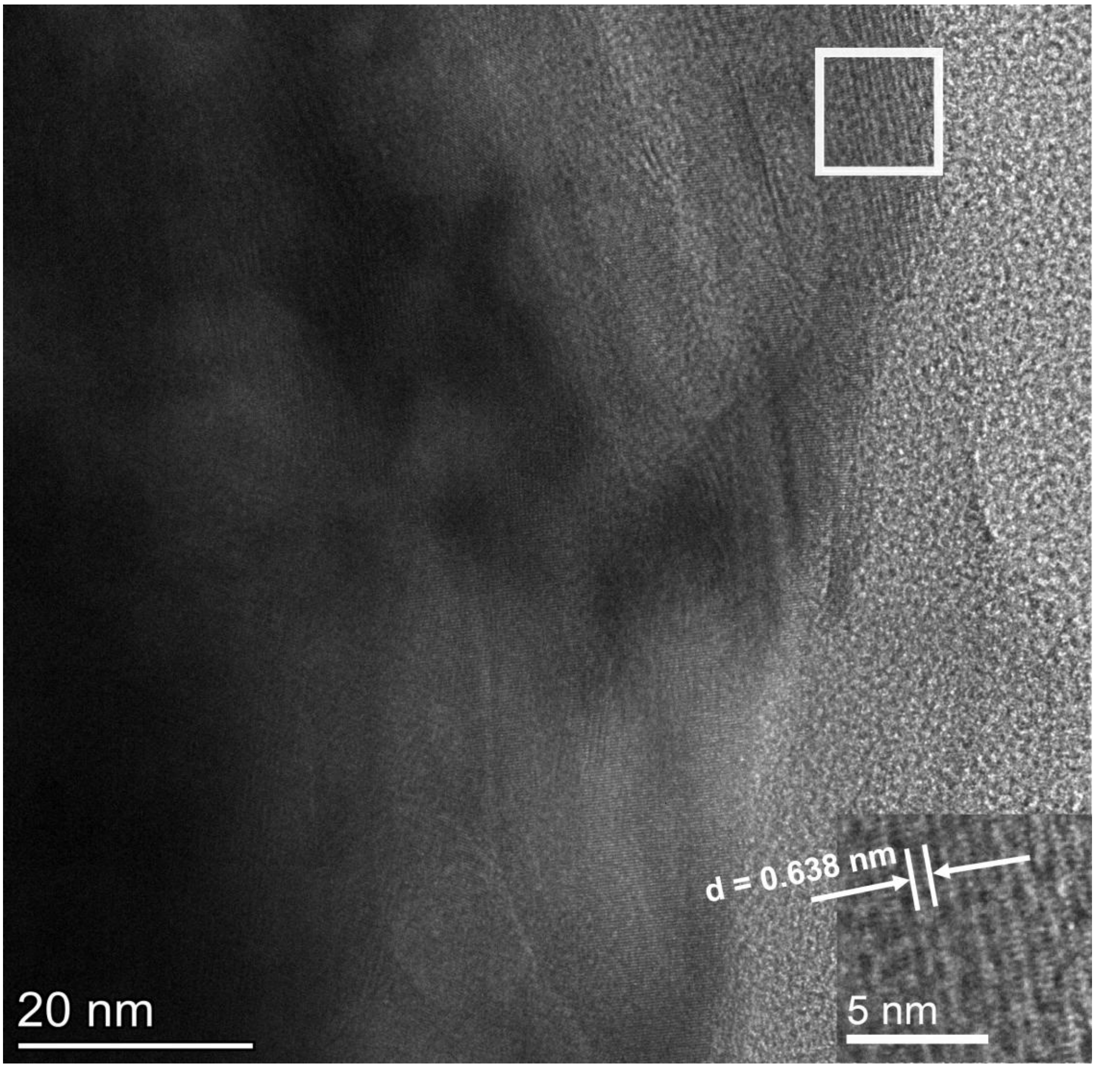

Figure S3. TEM images of MoS_2.

SI Note 6. XRD of Fe-MoS_2 with Different Fe Concentration

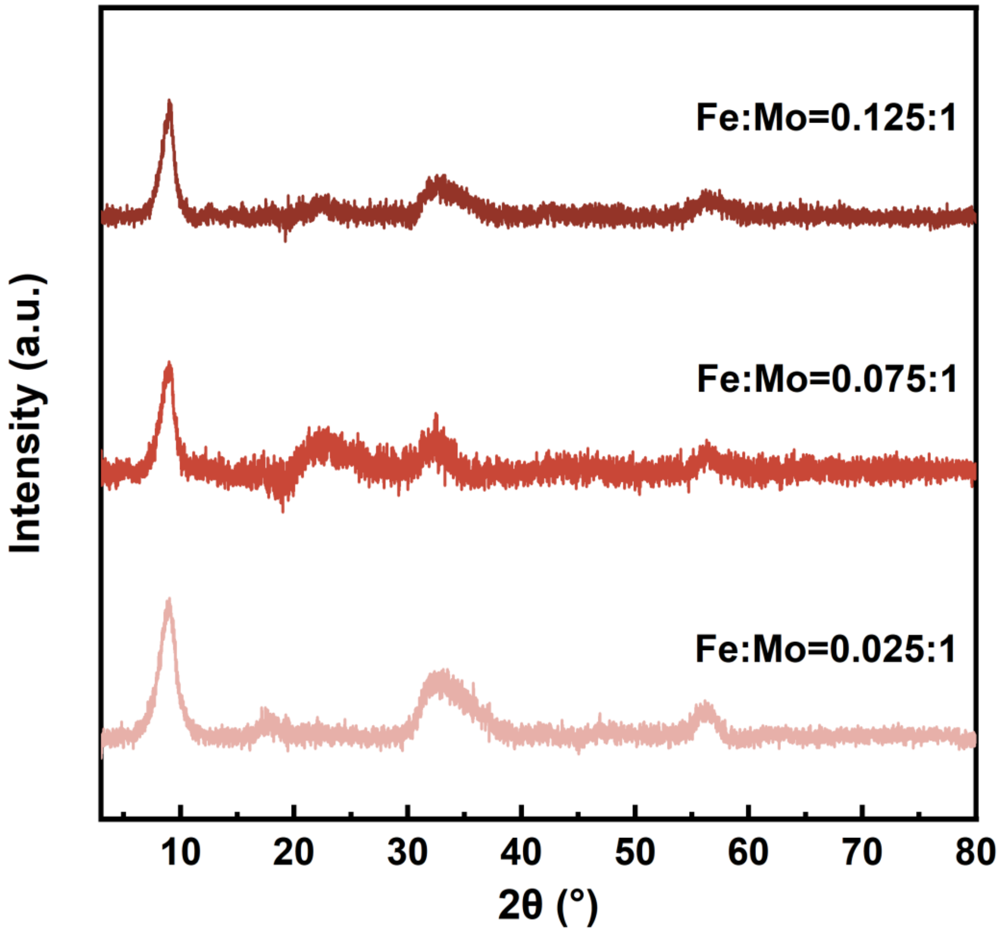

Figure S4. PXRD spectra of Fe-MoS_2 with Fe:Mo ratio changing from 0.025:1 to 0.125:1.

SI Note 7. XRD Spectra of M-MoS_2 and MoS_2 (M = Fe, Cu, Zn, Co, Mg, Ni, and Ru)

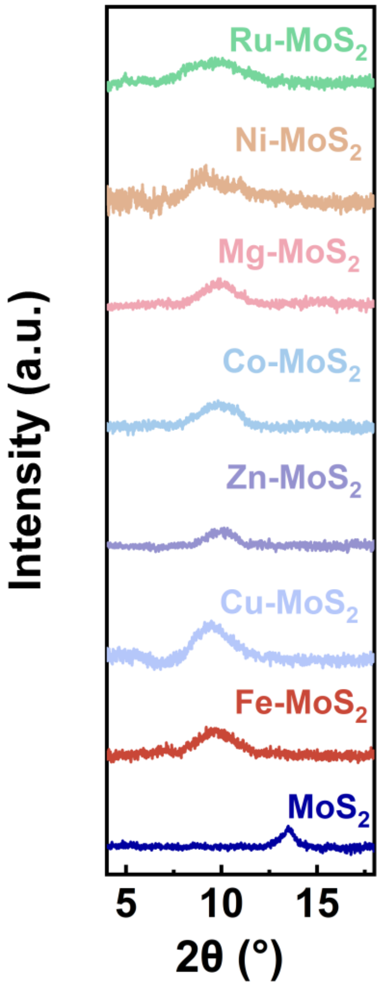

Figure S5. XRD spectra of M-MoS_2 and MoS_2 grown on carbon cloth. M = Fe, Cu, Zn, Co, Mg, Ni, and Ru.

Table S2. Interlayer spacings of MoS_2 and M-MoS_2 (M = Fe, Cu, Zn, Co, Mg, Ni, and Ru) determined by PXRD.

Dopant	(003) peak position (°)	d spacing (nm)
-	13.4	0.660
Fe	9.74	0.907
Cu	9.64	0.916
Zn	9.94	0.889
Co	9.89	0.893
Mg	10.0	0.883
Ni	9.36	0.944
Ru	9.41	0.938

SI Note 8. Raman Spectra of Fe-MoS_2, 1T MoS_2, and 2H MoS_2

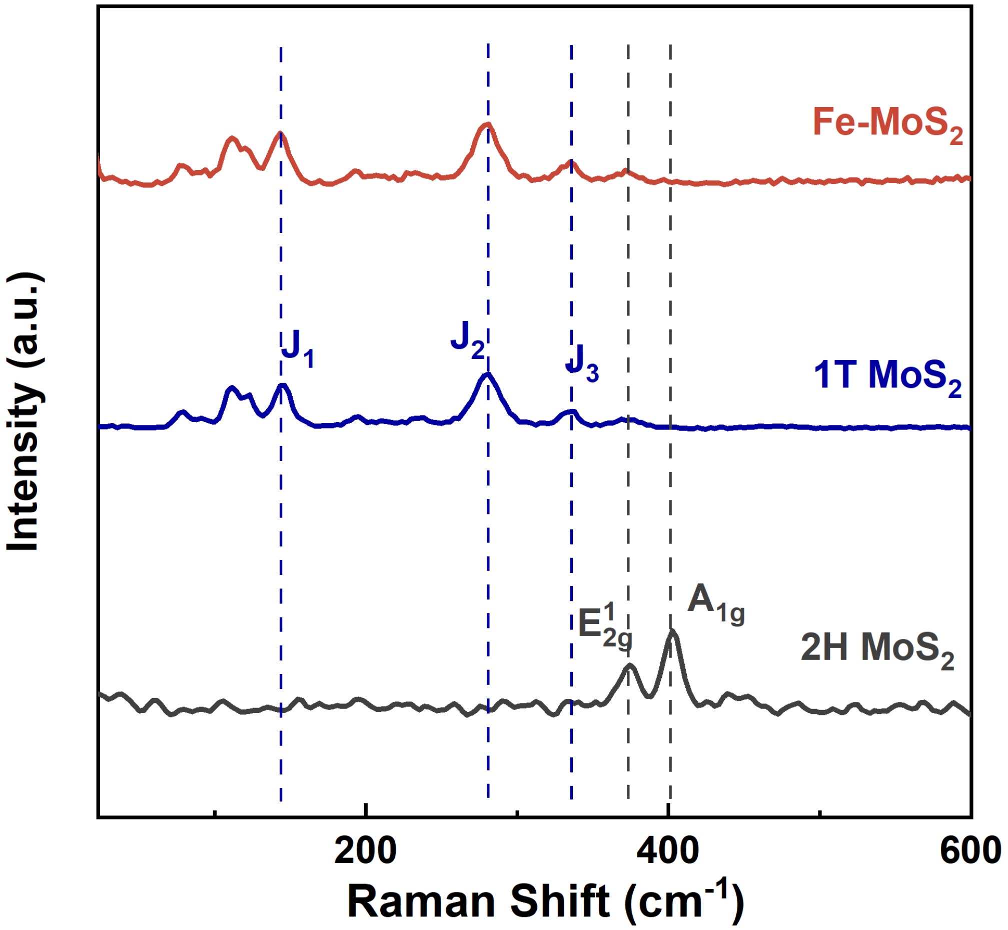

Figure S6. Raman spectra of Fe-MoS_2, 1T MoS_2, and 2H MoS_2.

SI Note 9. XAS Spectra of Fe-MoS_2

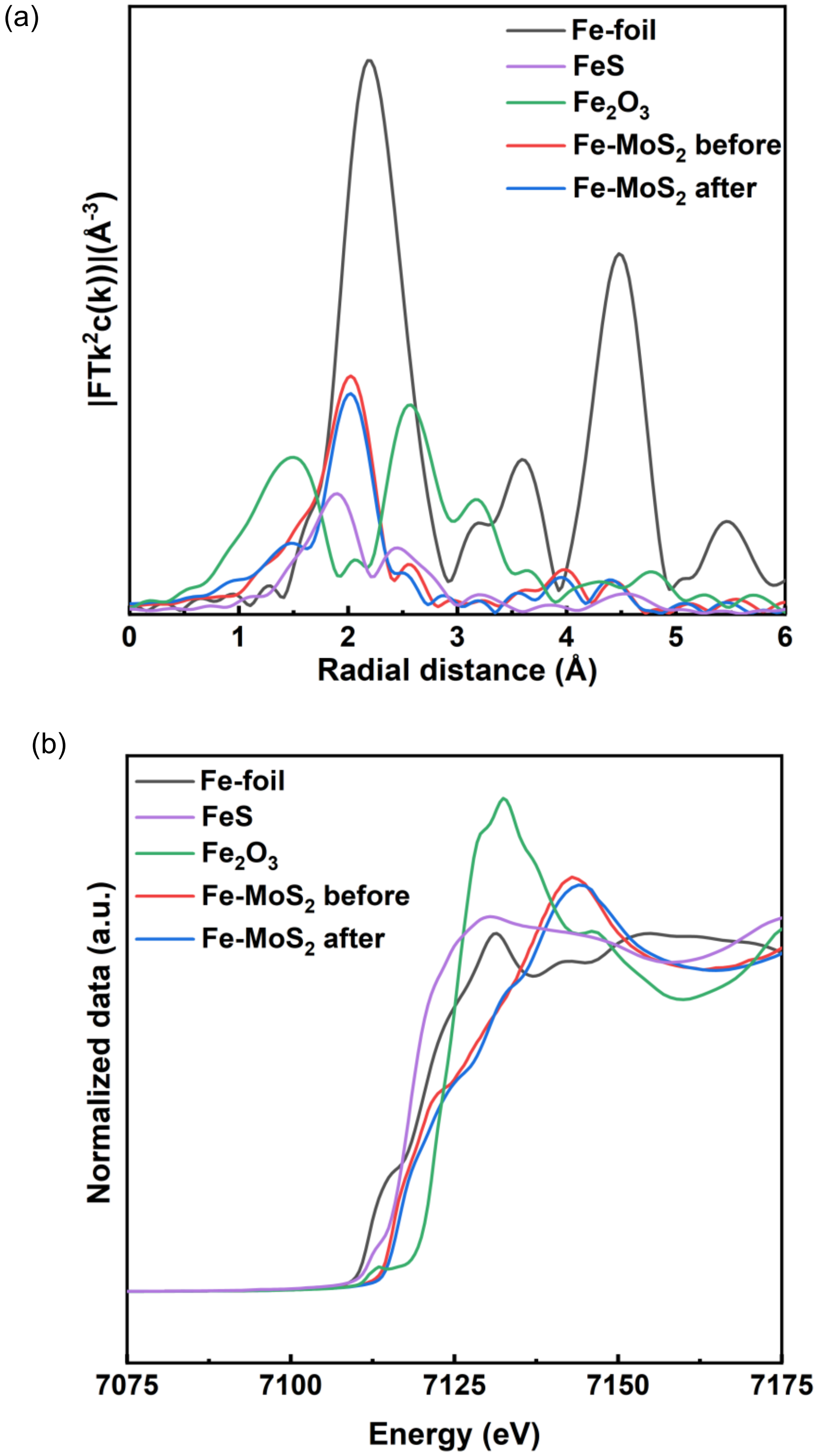

Figure S7. Fe K-edge (a) XANES spectra and (b) FT-EXAFS spectra of Fe-foil, FeS, Fe_2O_3, and Fe-MoS_2 before and after electrocatalytic C=N coupling.

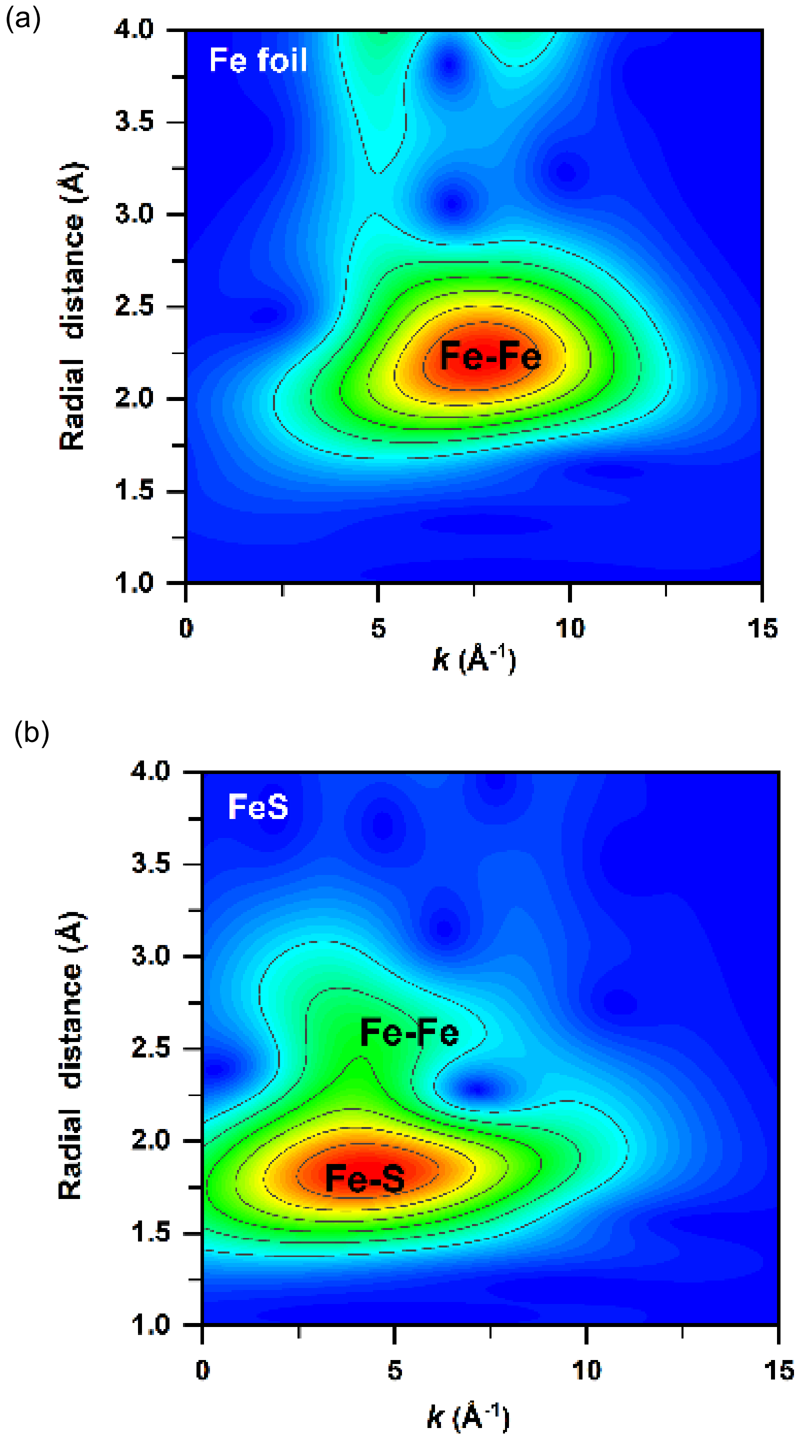
(a)
Fe foil
Fe-Fe
Radial distance (Å)
4.0
3.5
3.0
2.5
2.0
1.5
1.0
0
5
10
15
k (Å-1)
(b)
FeS
Fe-Fe
Fe-S
Radial distance (Å)
4.0
3.5
3.0
2.5
2.0
1.5
1.0
0
5
10
15
k (Å-1)

(c)

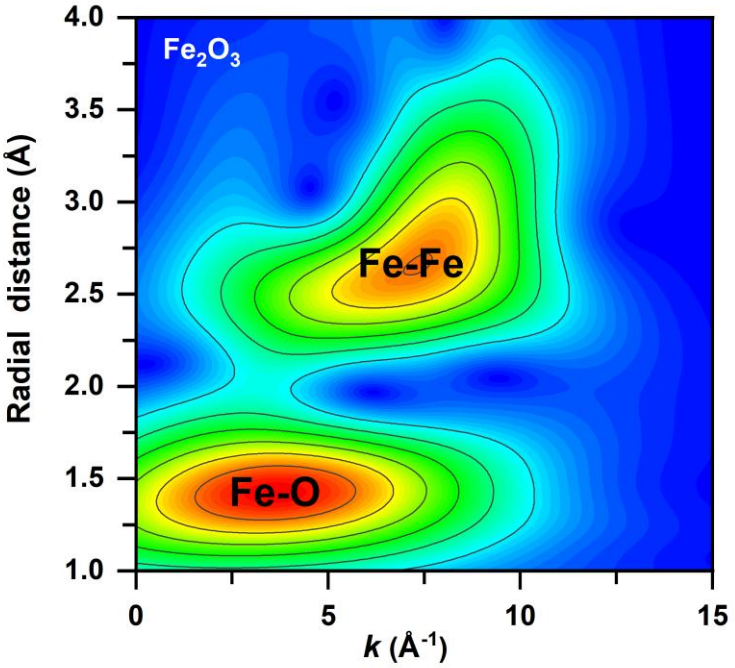

Fe2O3
Fe-Fe
Fe-O
Radial distance (Å)
k (Å-1)

(d)

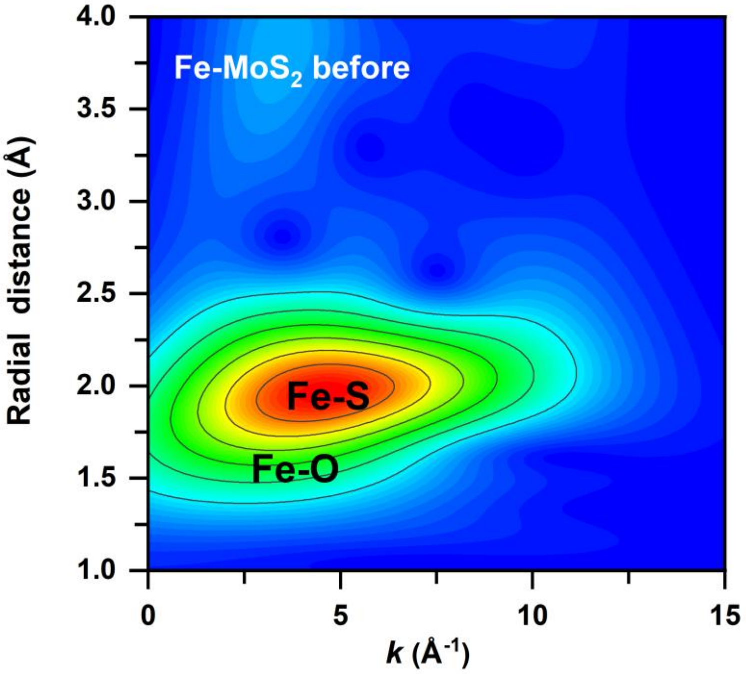

Fe-MoS2 before
Fe-S
Fe-O
Radial distance (Å)
k (Å-1)

(e)

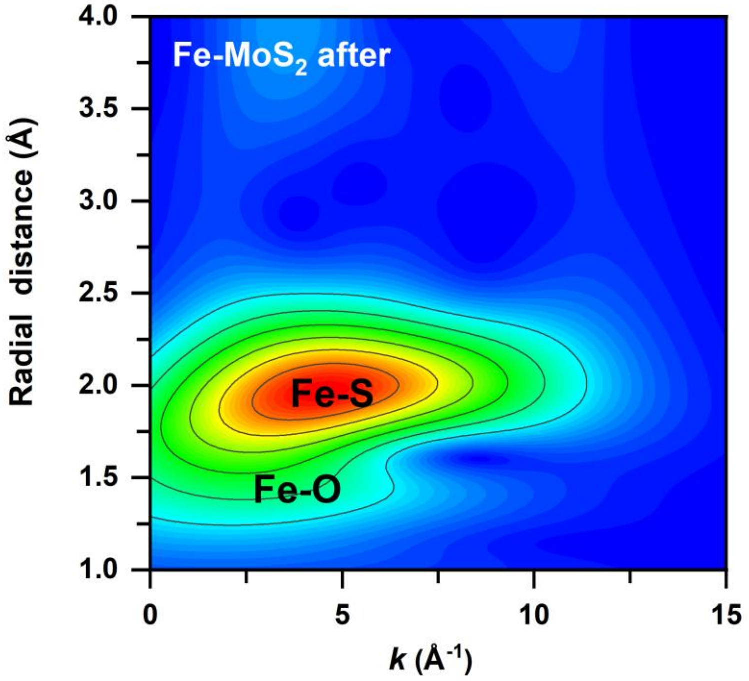

Figure S8. Wavelet-transform (WT) images of EXAFS data at Fe K-edge for (a) Fe foil, (b) FeS, (c) Fe_2O_3, (d) Fe-MoS_2 before electrolysis, and (e) Fe-MoS_2 after electrolysis.

SI Note 10. ^1H NMR of $H_2C=NOH$

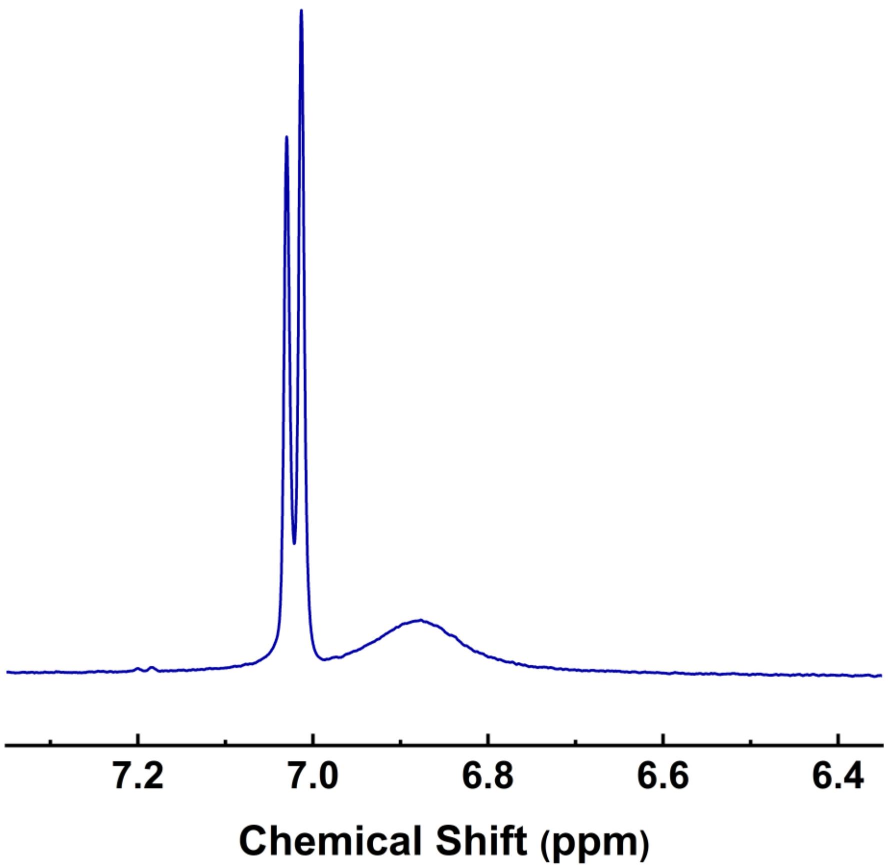

Figure S9. Detection of $H_2C=NOH$ in electrolyte solution by ^1H NMR.

SI Note 11. FE for $H_2C{=}NOH$, CH_3OH, NH_4^+, NH_2OH, and H_2 Using M-MoS_2 (M = Fe, Cu, Zn, Co, Mg, Ni, and Ru) as Catalysts

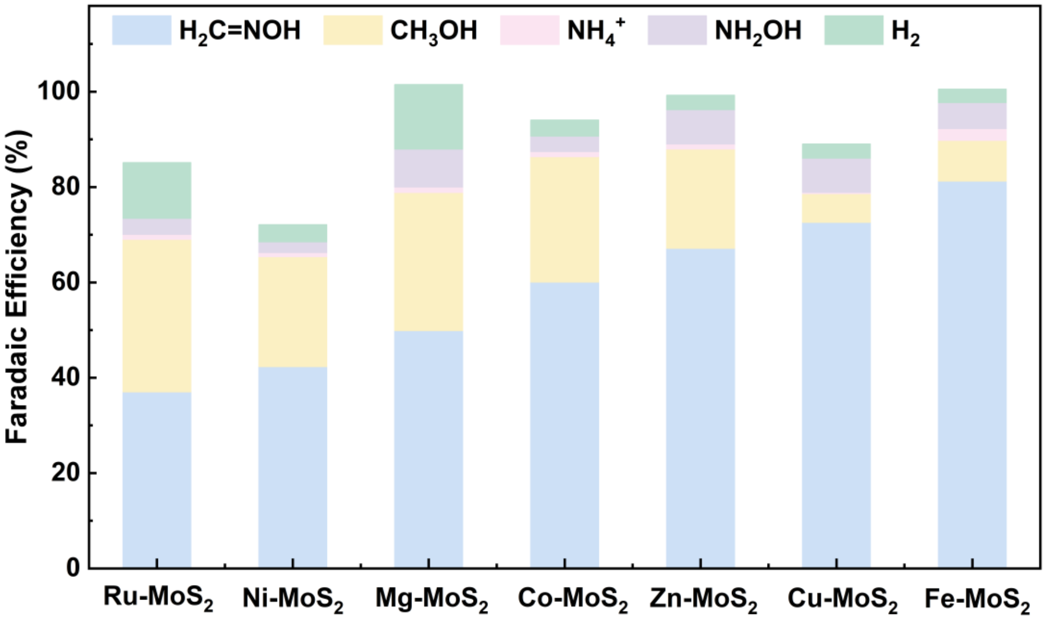

Figure S10. FE for $H_2C{=}NOH$, CH_3OH, NH_4^+, NH_2OH, and H_2 using M-MoS_2 with 0.5 M $NaNO_2$ and 0.5 M H_2CO in 0.1 M PBS (M = Fe, Cu, Zn, Co, Mg, Ni, and Ru).

SI Note 12. LSV Curves of Fe-MoS_2 (red) and MoS_2

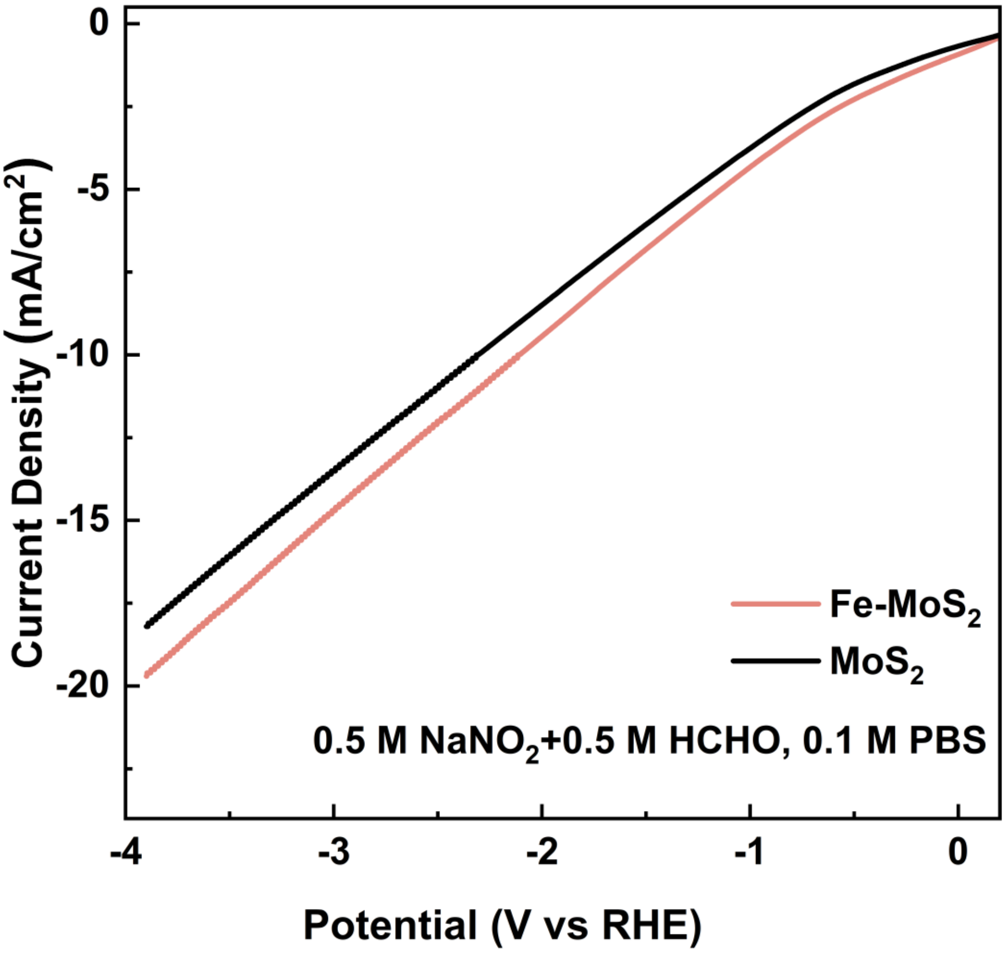

Figure S11. LSV curves of Fe-MoS_2 (red) and MoS_2 (black) in a 0.1 M PBS solution mixed with 0.5 M $NaNO_2$ and 0.5 M of H_2CO at a scan rate of 50 $mV{\cdot}s^{-1}$.

SI Note 13. YR for $H_2C=NOH$ Using Fe-MoS_2 as Electrocatalyst

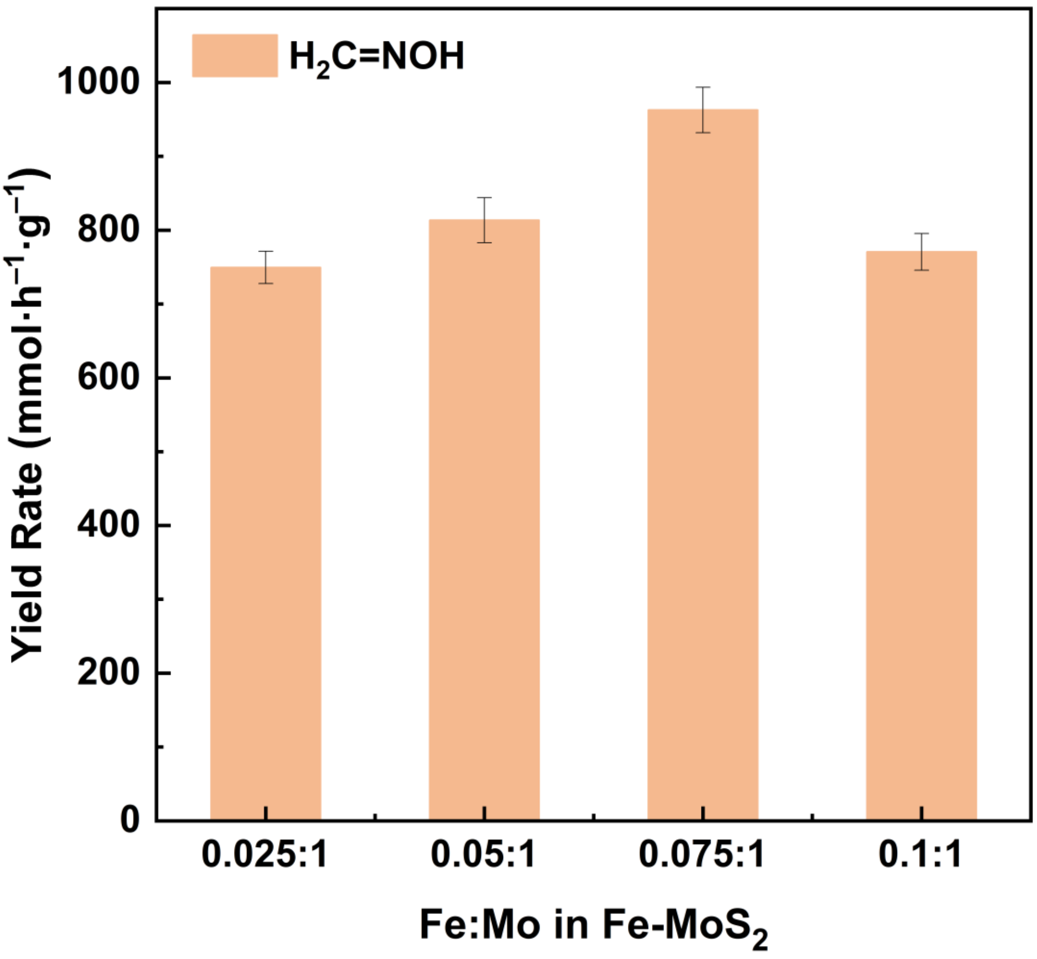

Figure S12. Effect of Fe:Mo ratio on the YR for $H_2C=NOH$ on Fe-MoS_2.

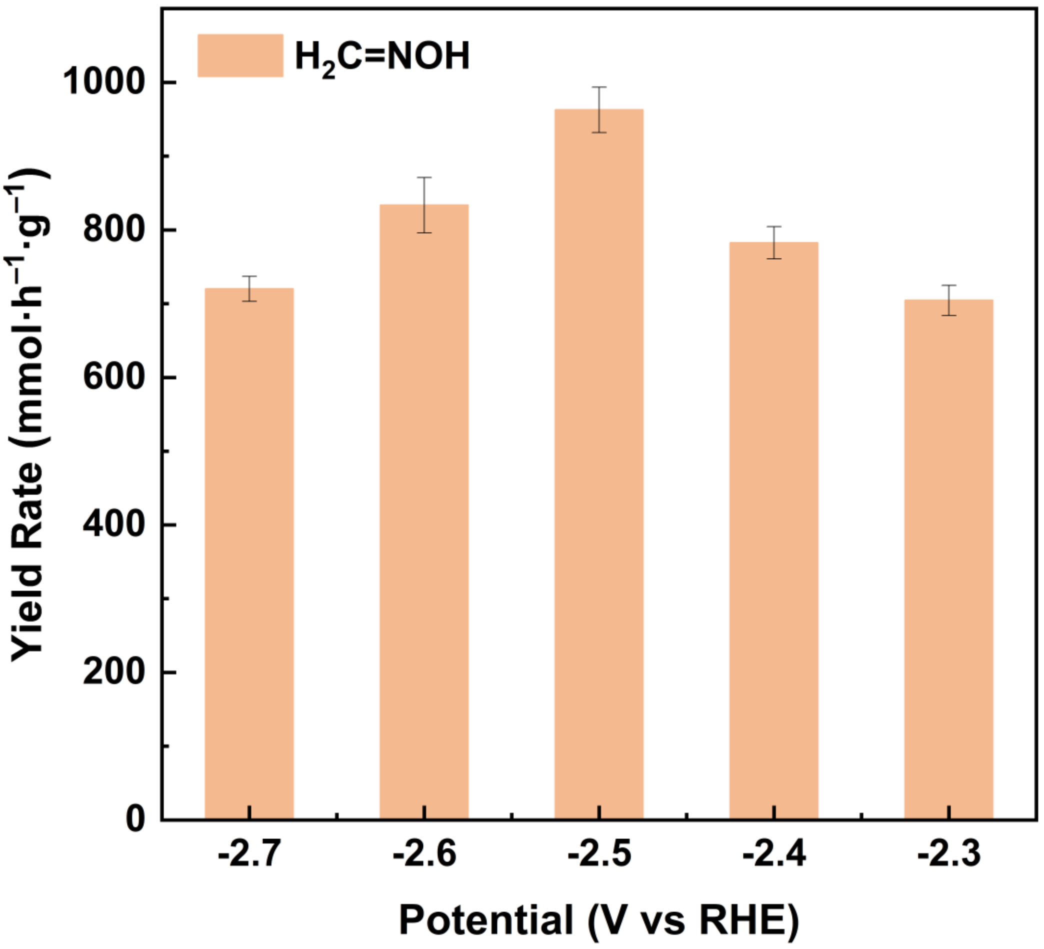

Figure S13. Potential-dependent YR of H_2C=NOH on Fe-MoS_2.

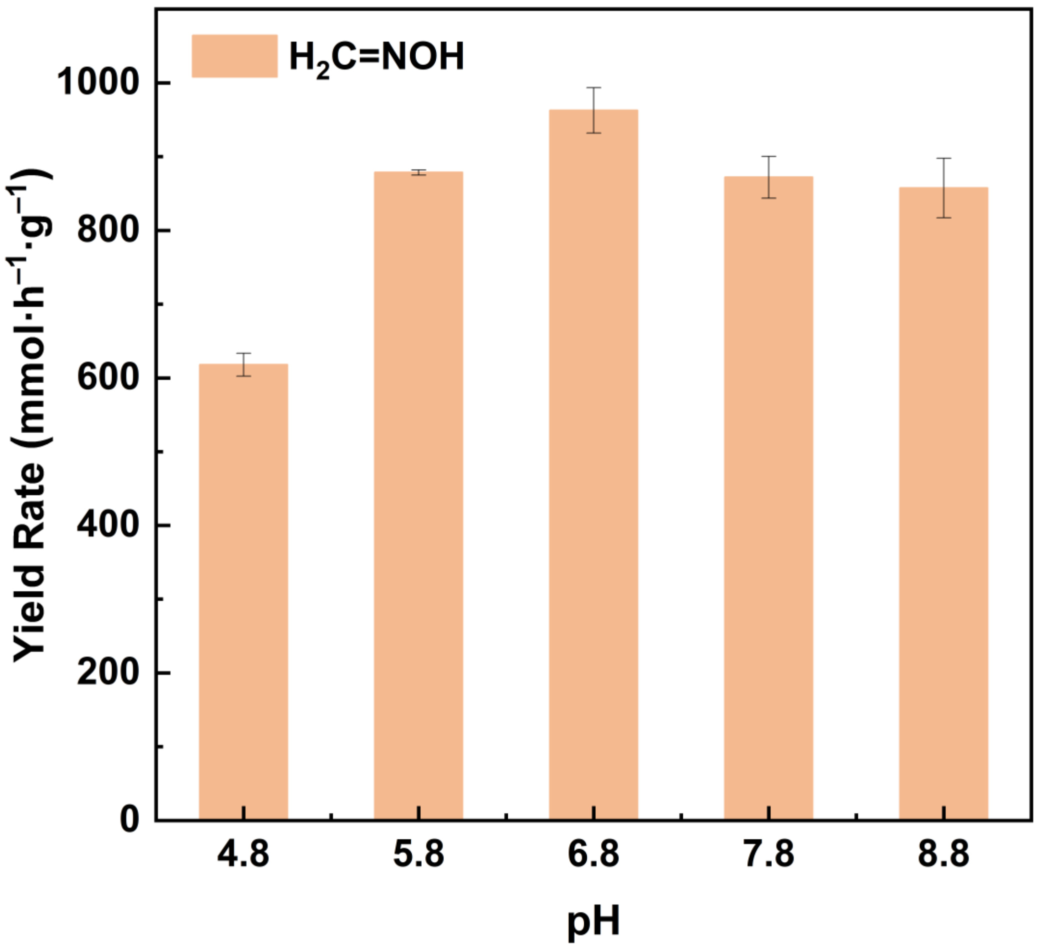

Figure S14. YR for $H_2C=NOH$ using Fe-MoS_2 with pH of electrolytes increasing from 4.8 to 8.8.

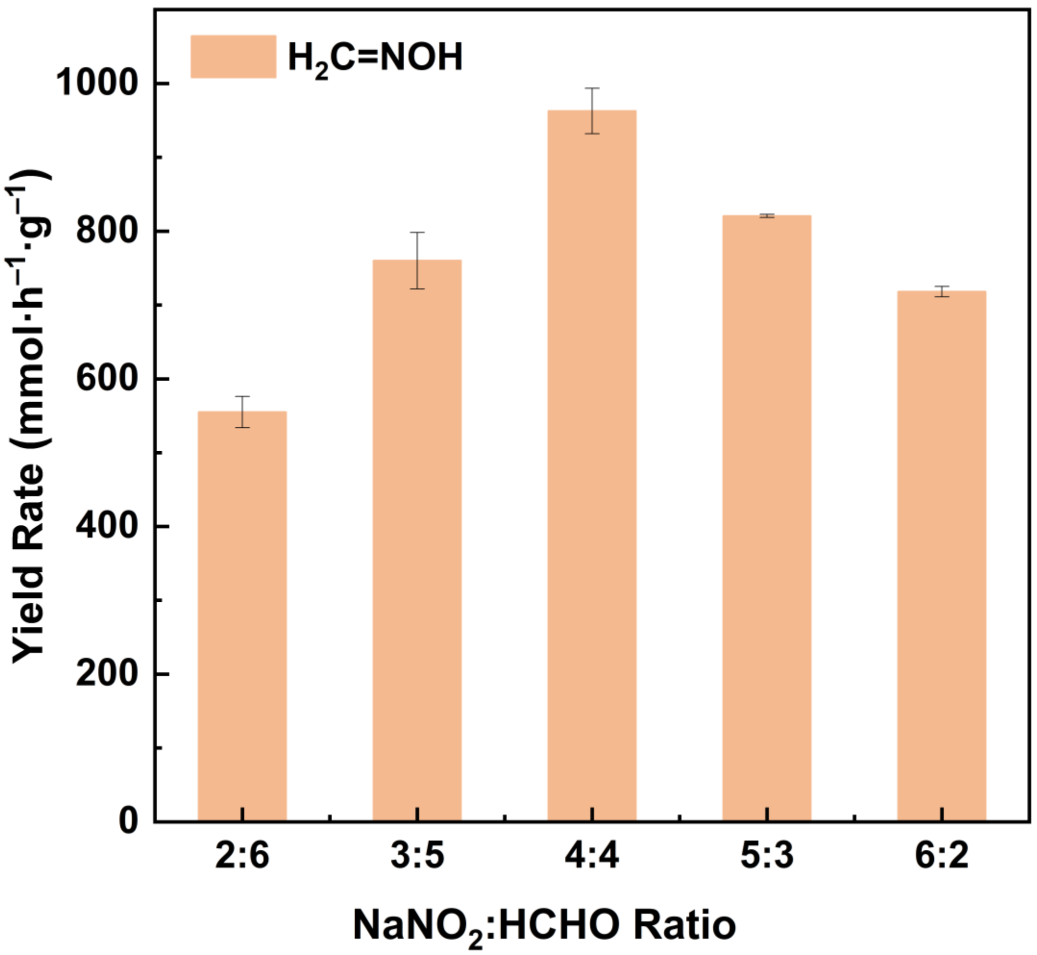

Figure S15. YR for $H_2C{=}NOH$ using Fe-MoS_2 with $NaNO_2$:H_2CO ratio changing from 6:2 to 2:6.

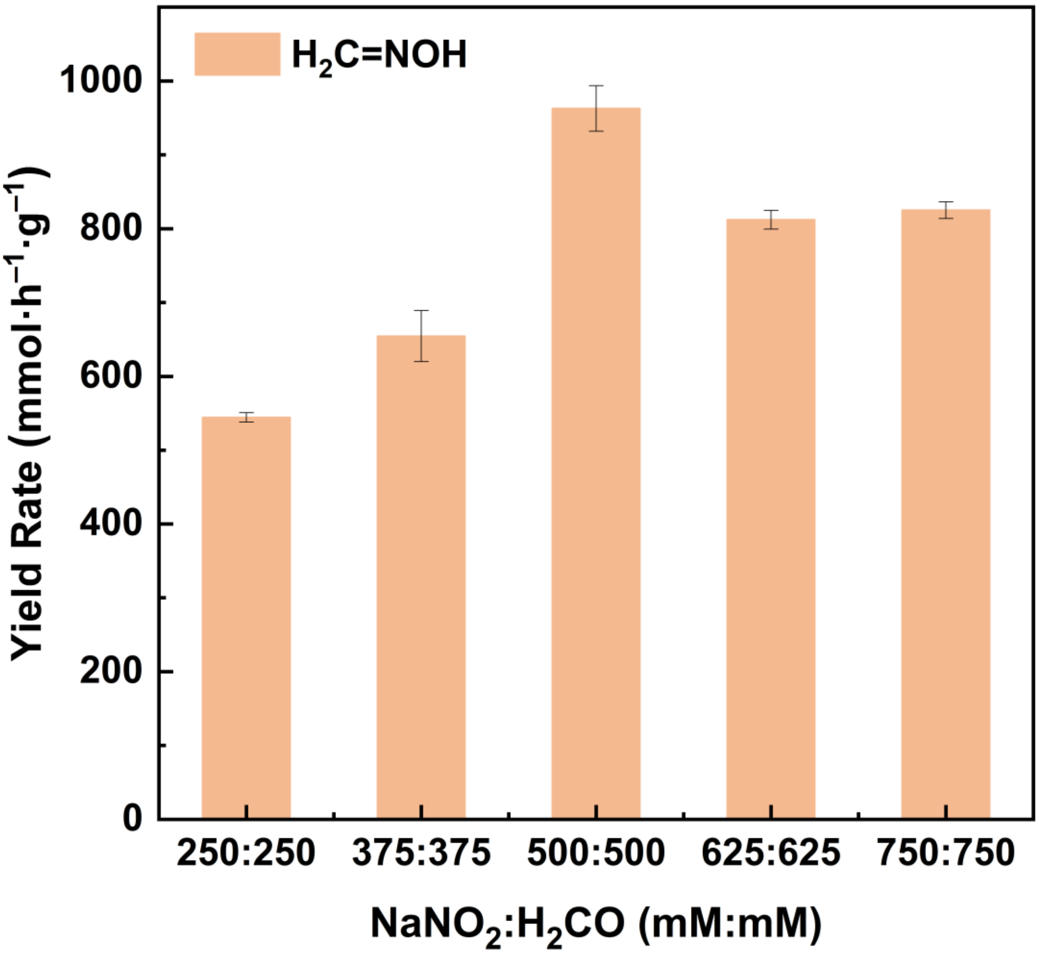

Figure S16. YR for $H_2C{=}NOH$ using Fe-MoS_2 with concentration of $NaNO_2$ or H_2CO changing from 250 mM to 750 mM.

SI Note 14. FE for Methanol, NH_4^+, and NH_2OH Using Fe-MoS_2 as Electrocatalyst

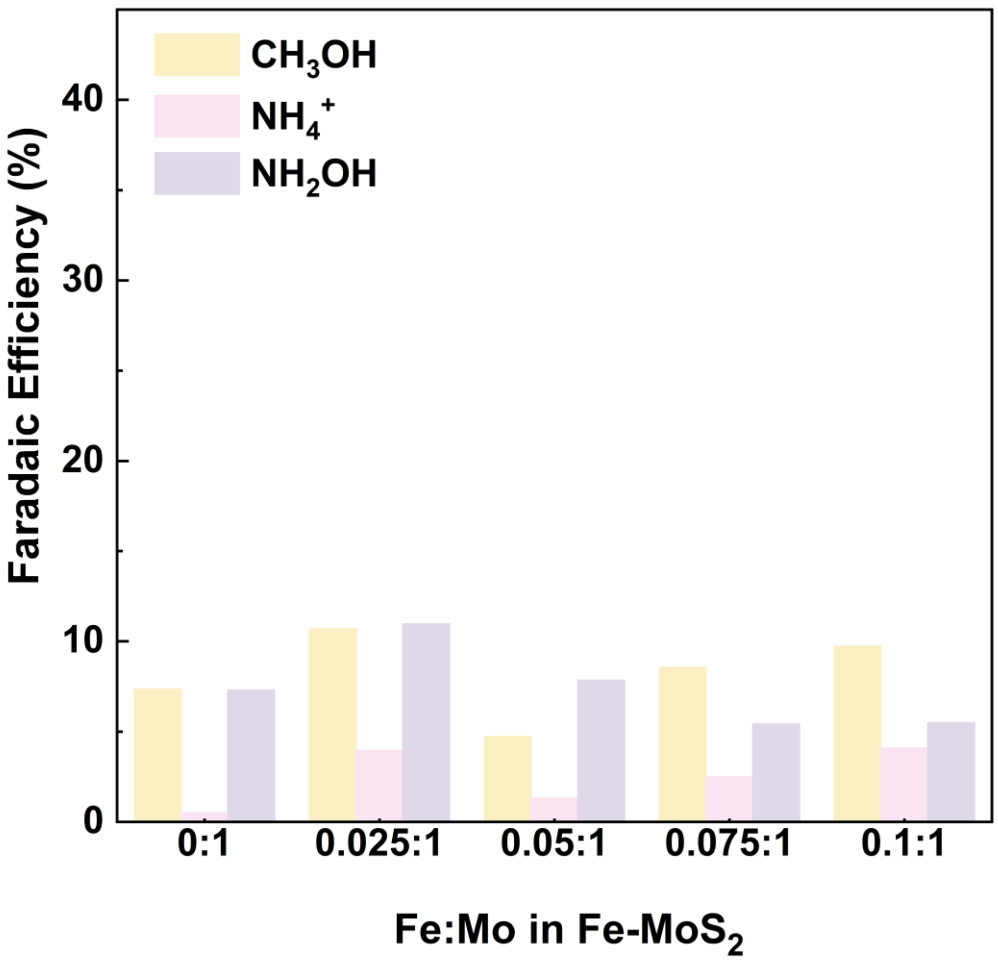

Figure S17. Effect of Fe:Mo ratio on the FE for methanol, NH_4^+, and NH_2OH on Fe-MoS_2 with Fe:Mo ratio changing from 0:1 to 0.1:1.

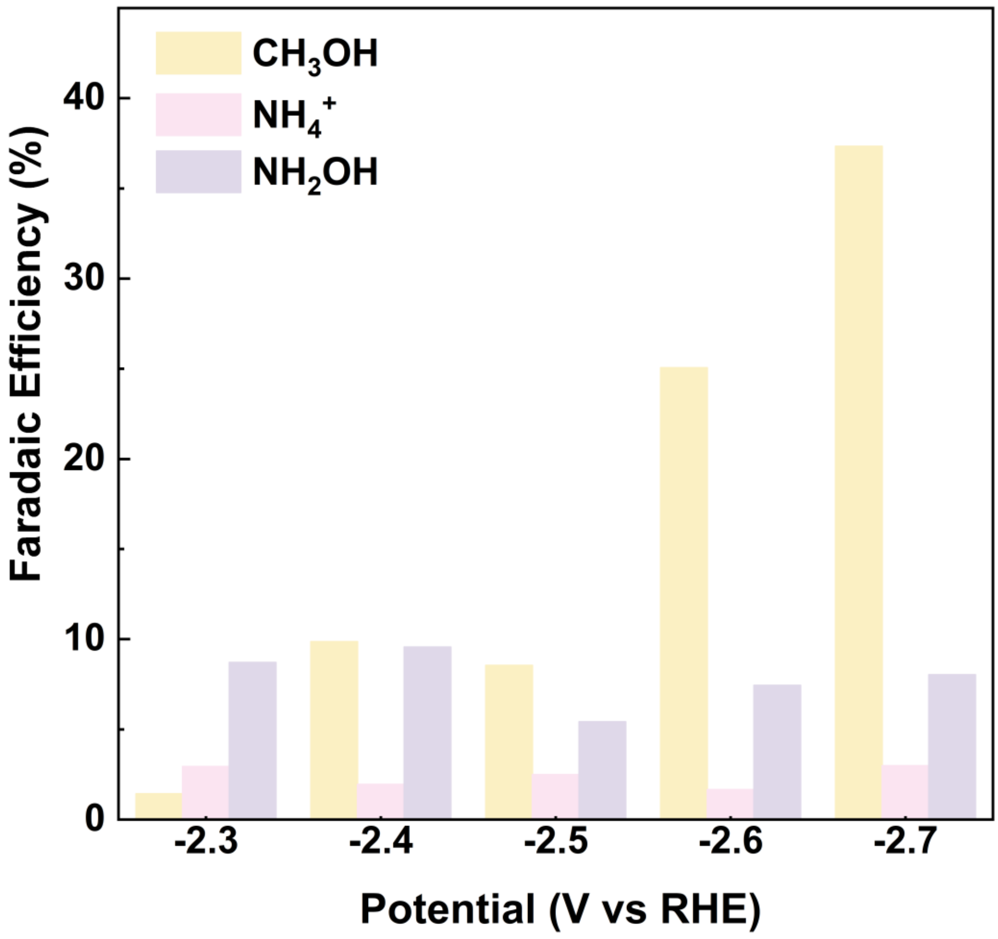

Figure S18. Potential-dependent FE for methanol, NH_4^+, and NH_2OH on Fe-MoS_2.

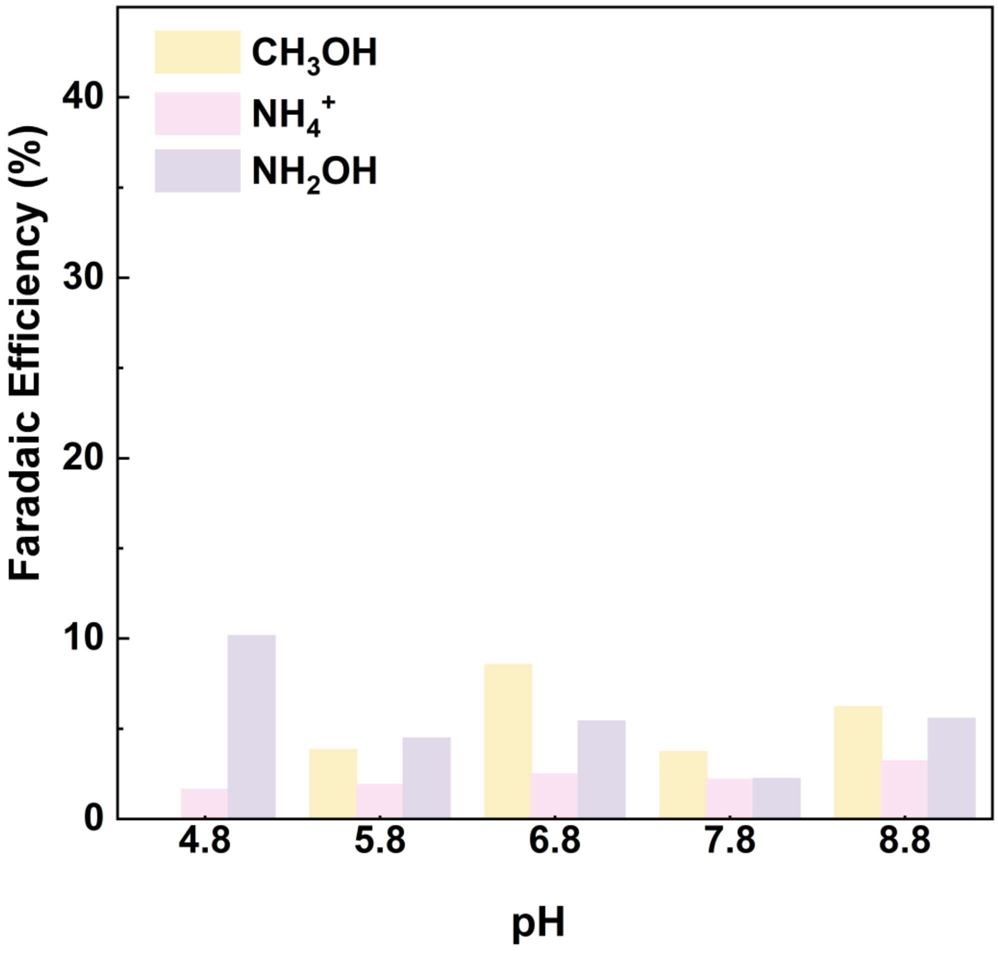

Figure S19. FE for methanol, NH_4^+, and NH_2OH using Fe-MoS_2 with pH of electrolyte solution increasing from 4.8 to 8.8.

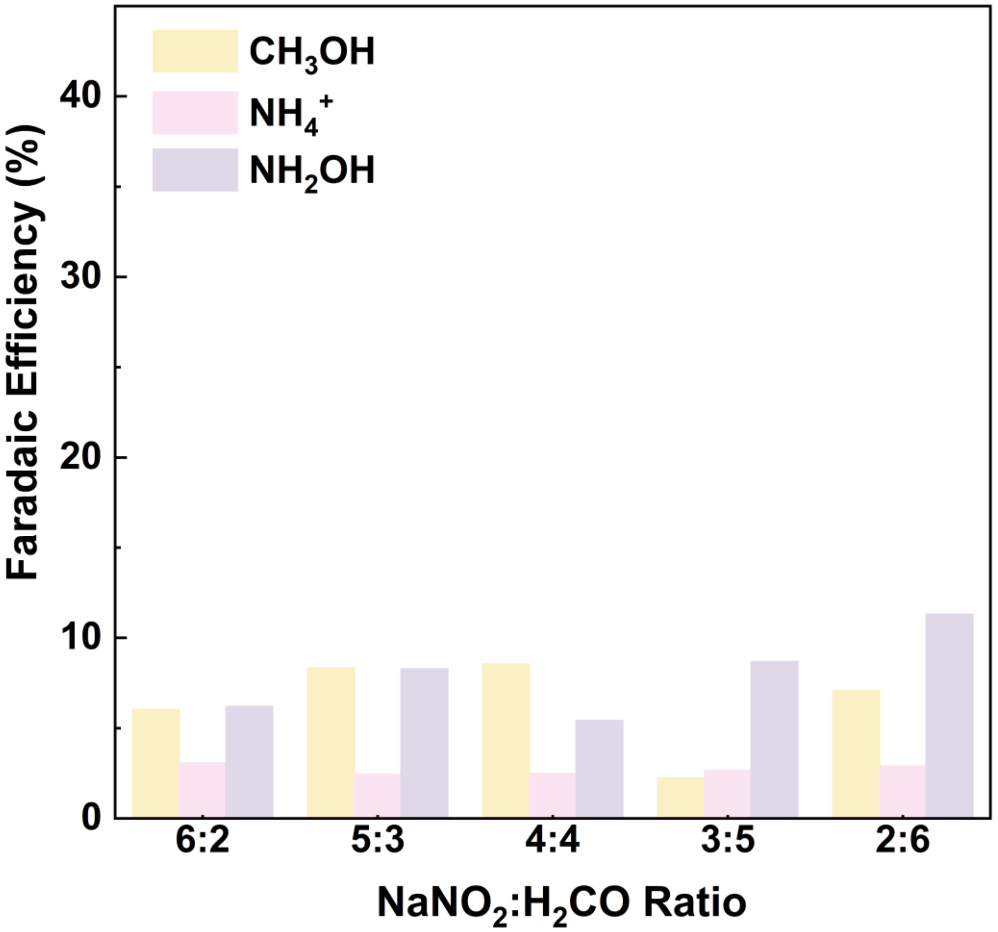

Figure S20. FE for methanol, NH_4^+, and NH_2OH using Fe-MoS_2 with $NaNO_2$:H_2CO ratio changing from 6:2 to 2:6.

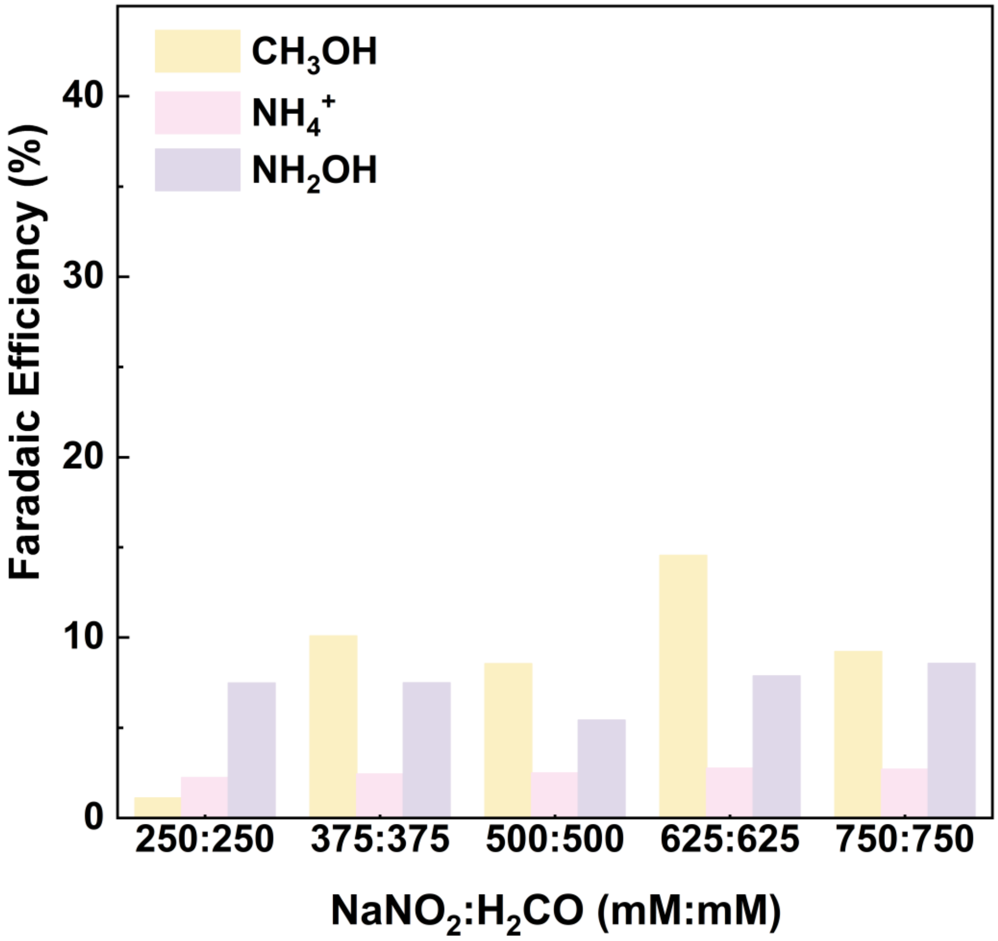

Figure S21. FE for methanol, NH_4^+, and NH_2OH using Fe-MoS_2 with concentration of $NaNO_2$ or H_2CO changing from 250 mM to 750 mM.

SI Note 15. YR for Methanol, NH_4^+, and NH_2OH Using Fe-MoS_2 as Electrocatalyst

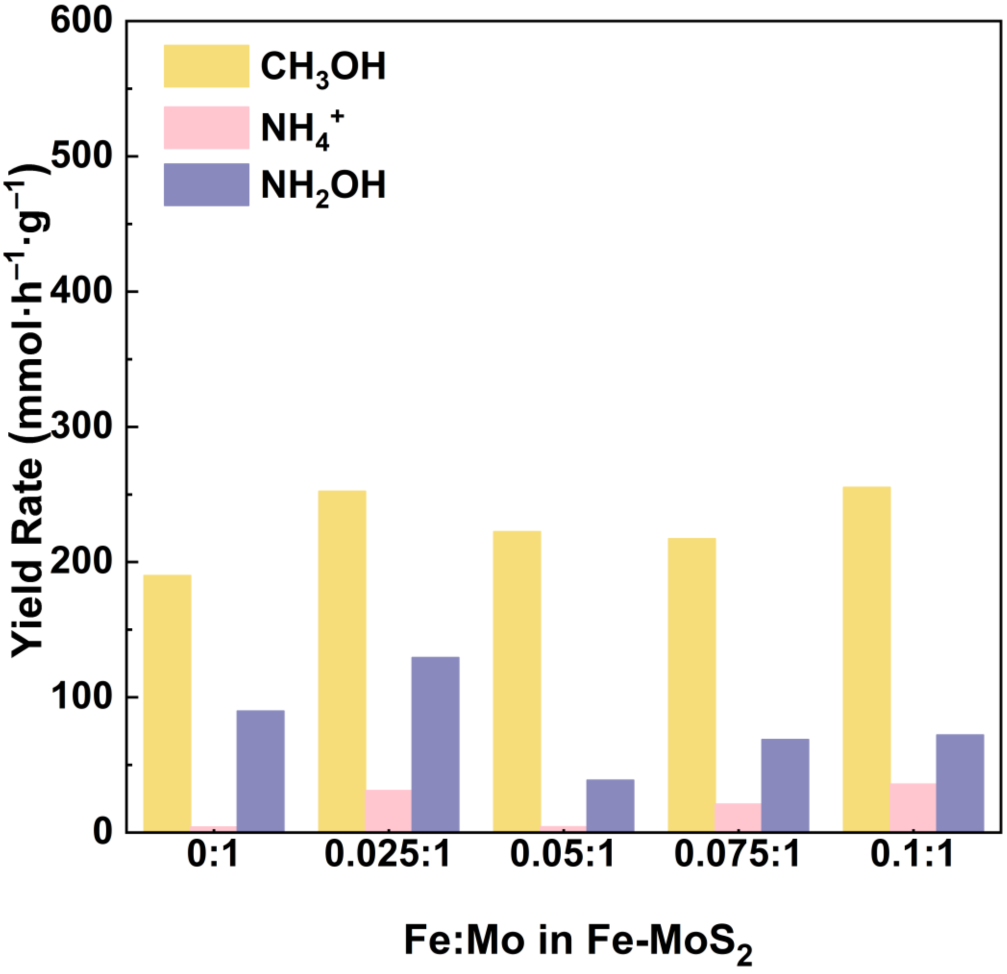

Figure S22. Effect of Fe:Mo ratio on the YR for methanol, NH_4^+, and NH_2OH on Fe-MoS_2 with Fe:Mo ratio changing from 0:1 to 0.1:1.

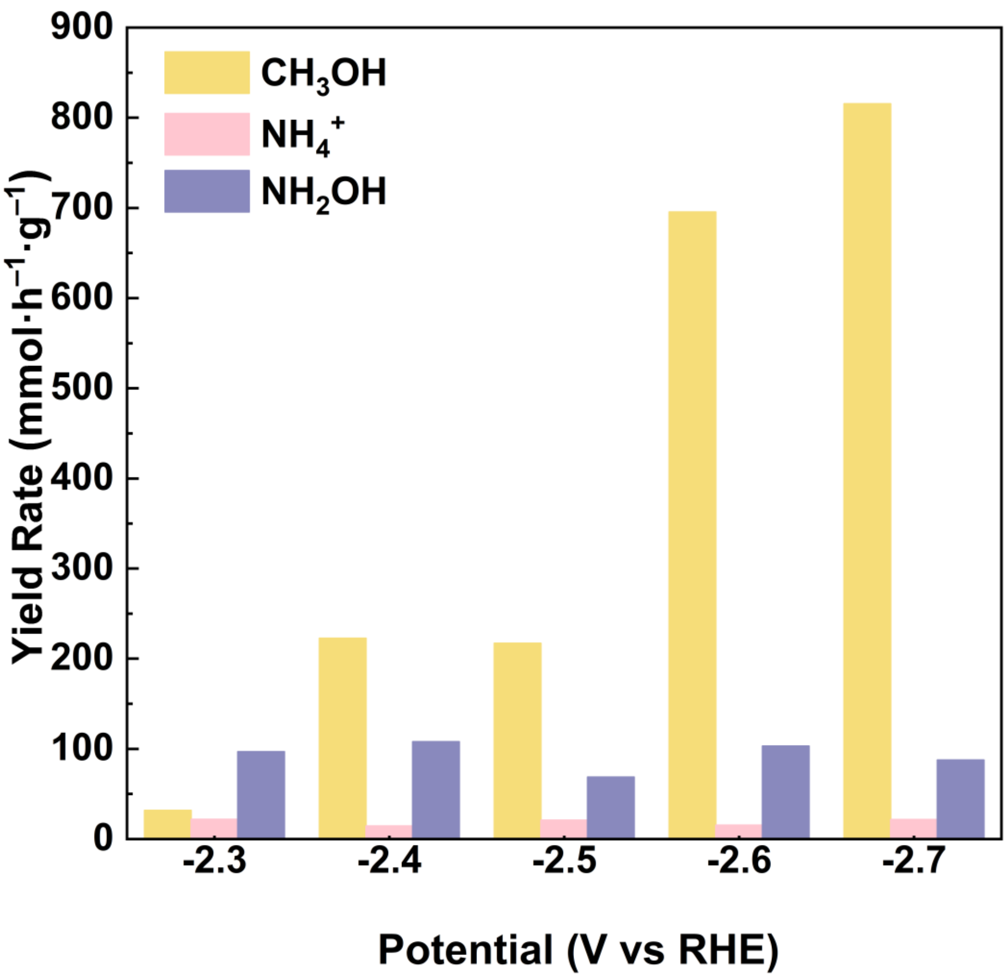

Figure S23. Potential-dependent YR for methanol, NH_4^+, and NH_2OH on Fe-MoS_2.

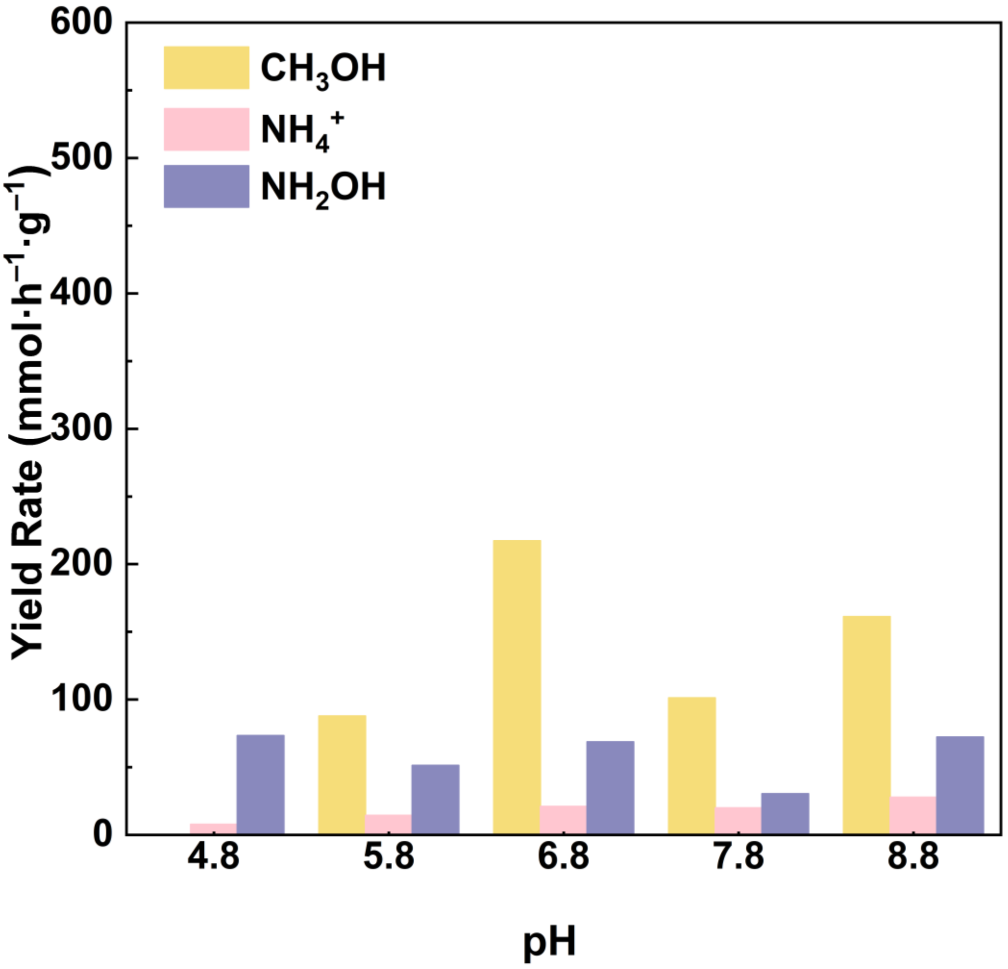

Figure S24. YR for methanol, NH_4^+, and NH_2OH using Fe-MoS_2 with pH of electrolytes increasing from 4.8 to 8.8.

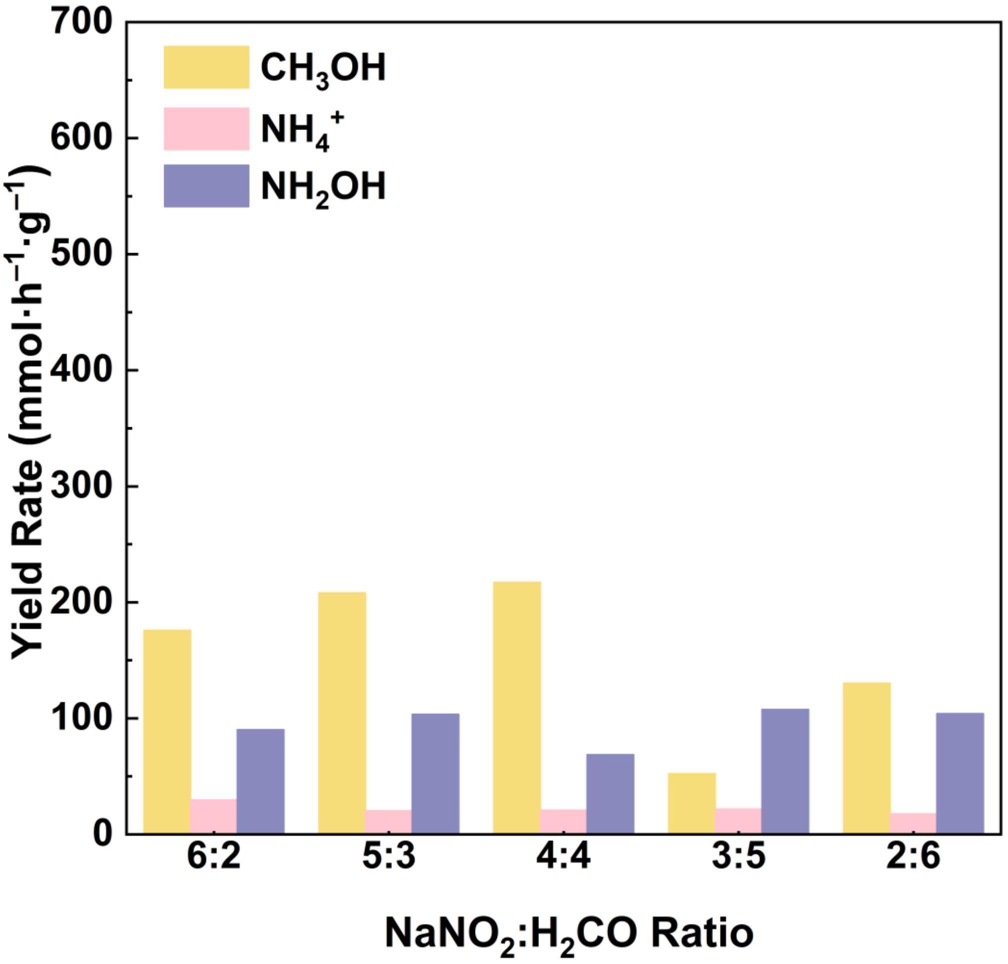

Figure S25. YR for methanol, NH_4^+, and NH_2OH using Fe-MoS_2 with $NaNO_2$:H_2CO ratio changing from 6:2 to 2:6.

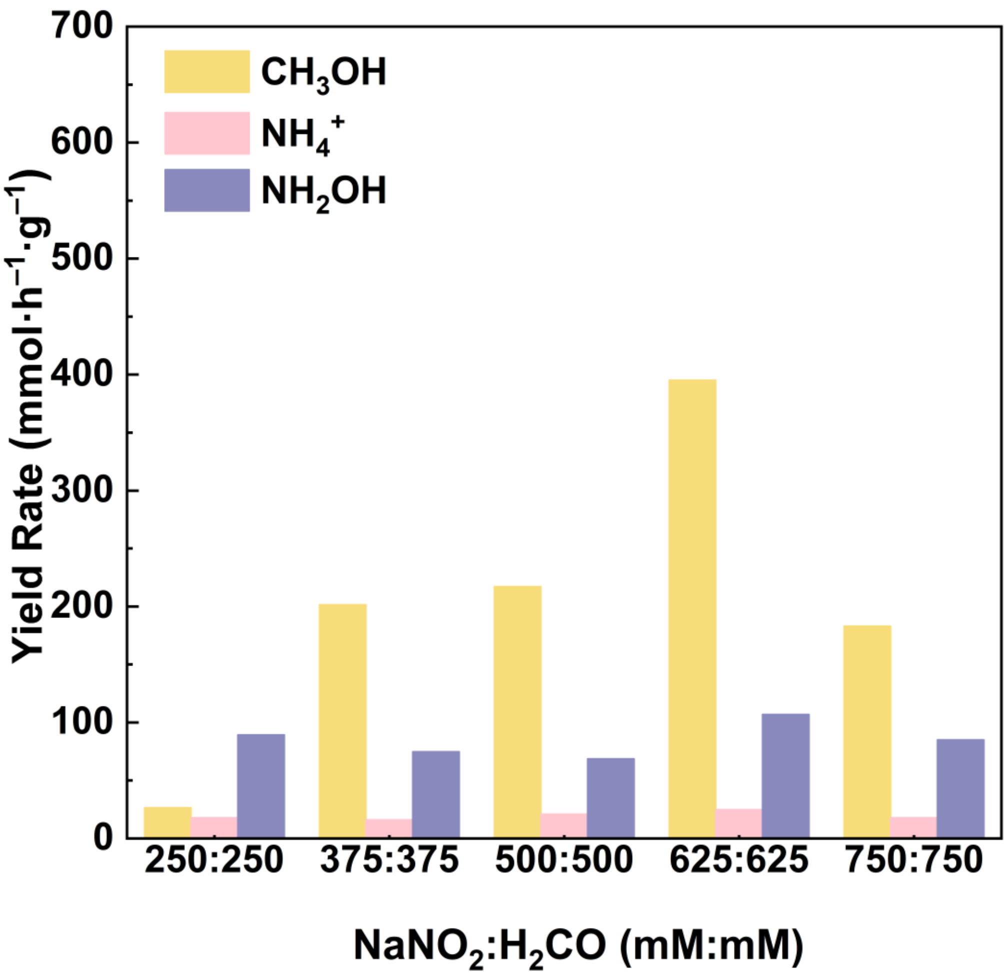

Figure S26. YR for methanol, NH_4^+, and NH_2OH using Fe-MoS_2 with concentration of $NaNO_2$ or H_2CO changing from 250 mM to 750 mM.

When the ratio of Fe dopant and Mo increases from 0:1 to 0.025:1, the FE and YR for methanol and N-species increase, which is plausibly attributed to the enhanced electrocatalytic performance with Fe dopant for H_2CO reduction and NO_2RR. Further increasing the Fe dopant concentration led to a decline in selectivity and activity, likely due to the suppressed electrosynthesis of $H_2C=NOH$. When the applied potentials is changed negatively, the FE and YR for methanol rise, whereas those for N-species decrease. This trend may be due to the stronger adsorption of neutral molecules compared to anionic species, such as NO_2^-, at the cathode under more negative potentials. When the pH value of the electrolyte changes from 4.8 to 8.8, the FE and YR for methanol has a volcano-shaped trend, which is ascribed to the high selectivity

of HER in acidic solution and the limited coverage of surface H* under basic solution. In contrast, the generation of N-species is more favorable in the acidic solution, plausibly due to the high coverage of surface H*. By adjusting the ratio of C- and N-sources, the highest selectivity of N-species is observed when the ratio of H_2CO and NO_2^- is 2:6, consistent with the high adsorption and concentration of NO_2^- on Fe-MoS_2. Only when the ratio of H_2CO and NO_2^- is 1:1, the selectivity of NH_2OH from NO_2RR is lower than that of methanol from H_2CO reduction reaction, indicating significant inhibition for NO_2RR caused by the C=N coupling step. When the concentration of both C- and N-sources increase, the FE and YR for methanol, NH_4^+, and NH_2OH reaches highest when the concentration of H_2CO and NO_2^- is 625 mM, owing to the high concentration on the surface of Fe-MoS_2. When the concentration increases from 375 mM to 500 mM, the FE and YR decrease which potentially results from the suppression of competing H_2C=NOH electrosynthesis.

SI Note 16. Stability test of Fe-MoS_2 as Electrocatalyst

(a)

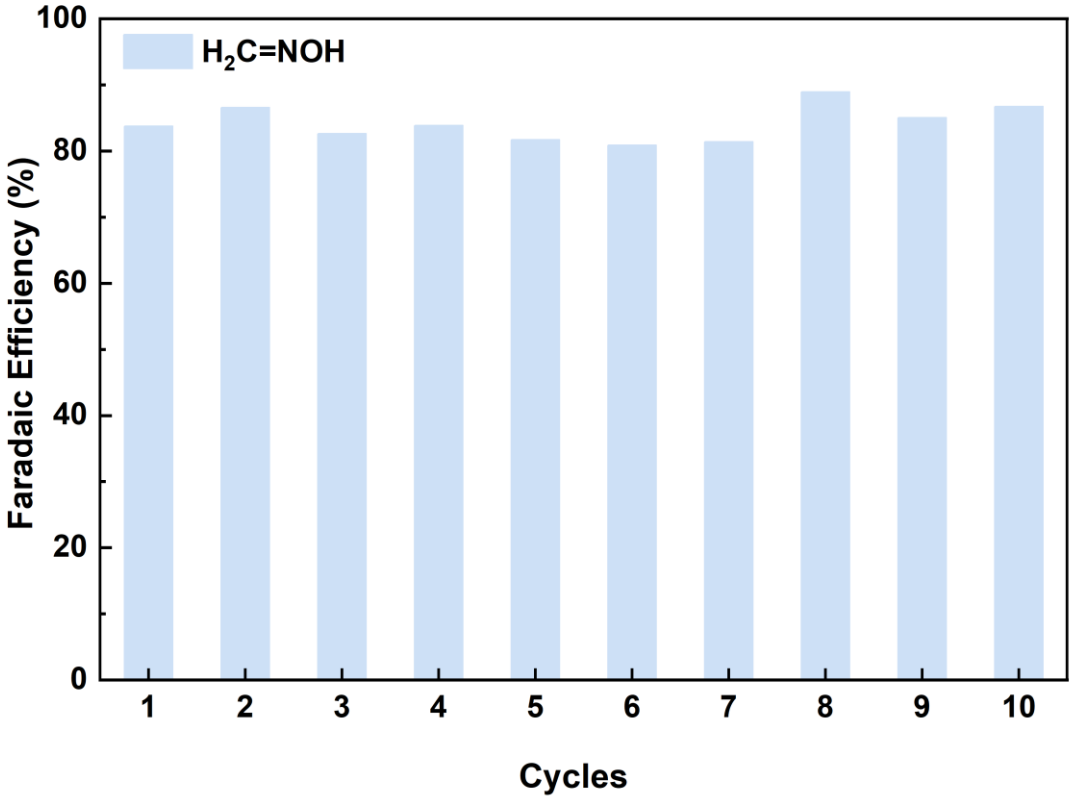

(b)

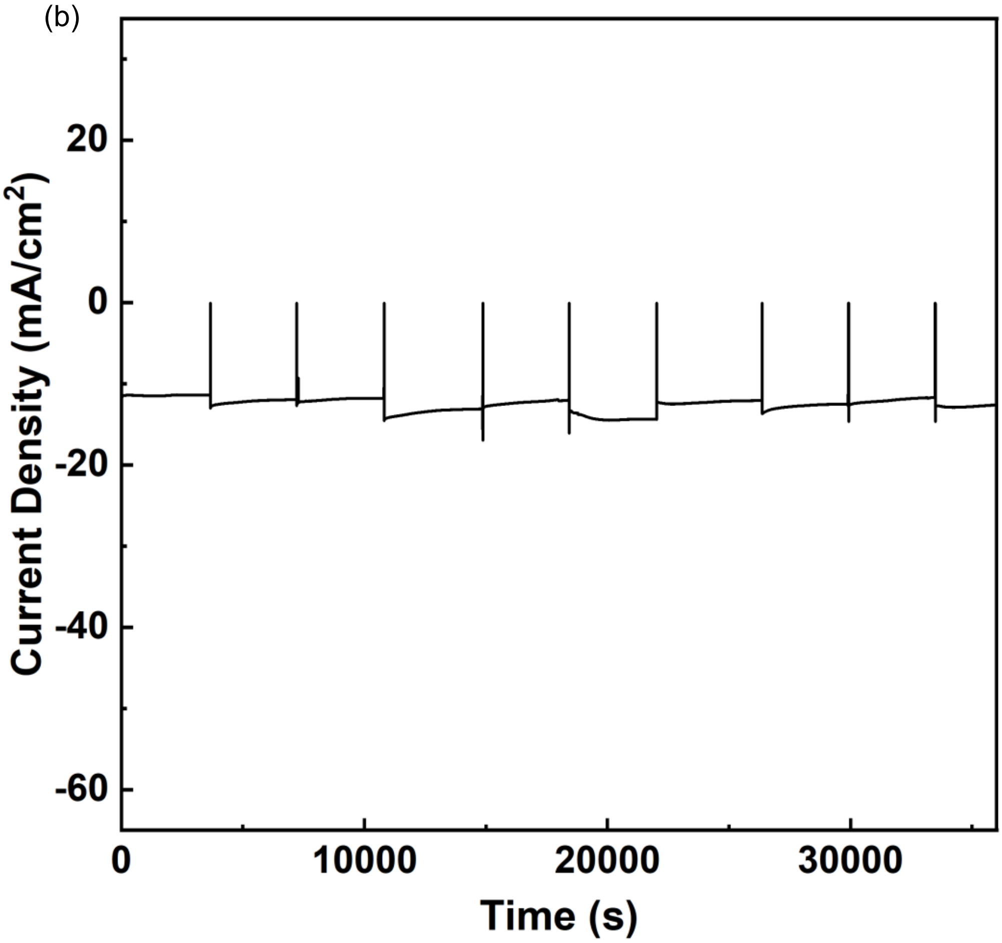

Figure S27. (a) FE for $H_2C=NOH$ during 10-continuous cycle test using Fe-MoS_2. (b) Current density during 10-continuous cycle test using Fe-MoS_2. The discontinuities are caused by replenishing the H-cell with fresh electrolyte solutions.

SI Note 17. ^{1}H NMR Spectra and [2]D NMR Spectra of the Reaction Solutions in Cross Isotopic Labelling Experiments

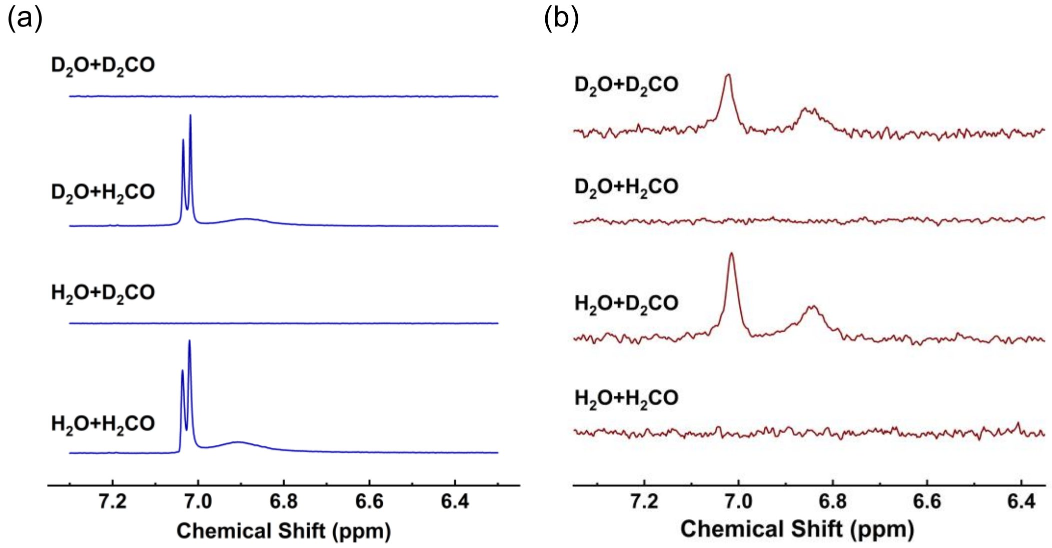

Figure S28. Detection of electrocatalytically synthesized $H_2C{=}NOH$ by (a) ^1H NMR and (b) ^2D NMR using protonated and deuterated solvents and reactants: (i) H_2O + H_2CO, (ii) H_2O + D_2CO, (iii) D_2O + H_2CO, and (iv) D_2O + D_2CO.

SI Note 18. ^{1}H NMR Spectra of the Reaction Solutions in reactants control experiments

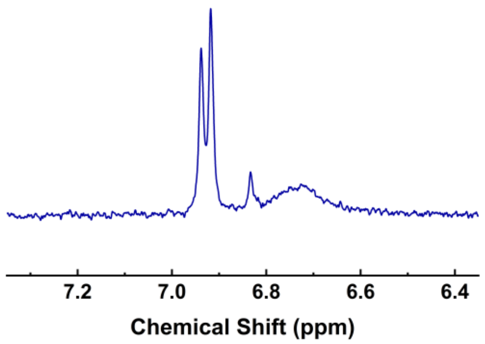

Figure S29. ^{1}H NMR spectra of the reaction solution in the control experiment using NO and H_2CO as reactants.

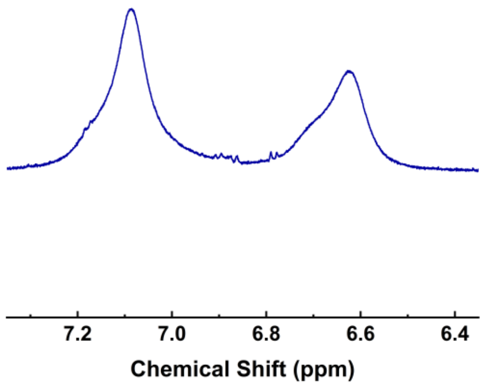

Figure S30. ^{1}H NMR spectra of the reaction solution in the control experiment using NH_2OH and H_2CO as reactants.

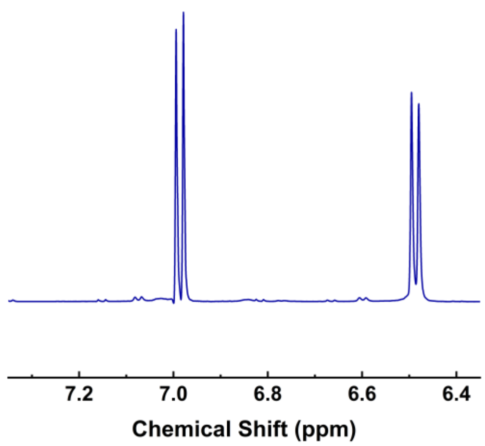

Figure S31. ^{1}H NMR spectra of the reaction solution in the control experiment using NH_2OH and H_2CO as reactants without applied potential nor current.

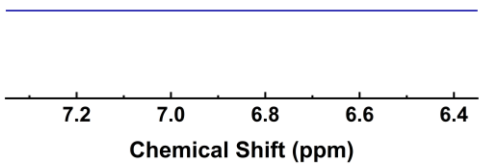

Figure S32. ^{1}H NMR spectra of the reaction solution in the control experiment using NH_4^+ and H_2CO as reactants.

SI Note 19. ^1H NMR Spectra of the Reaction Solutions in H_2 Control without Applied Potential nor Current

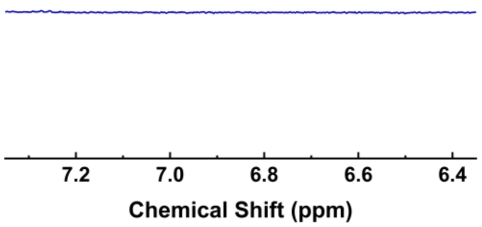

Figure S33. ^1H NMR spectra of the reaction solution in the control experiment with no electrochemical potential applied (i.e. no electrical current passed) before and after 1 hour reaction in the presence of H_2.

SI Note 20. Entire in-situ IR Spectra of $H_2C=NOH$ Electrosynthesis on Fe-MoS_2

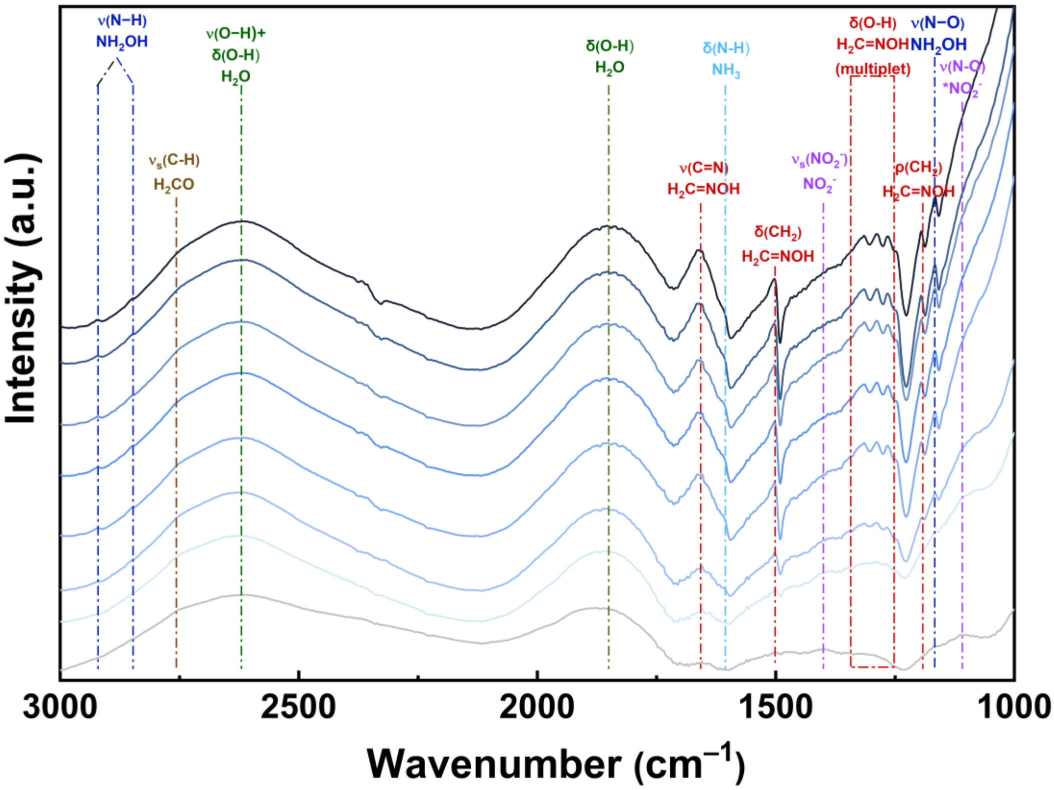

Figure S34. ATR-FTIR spectra for the electrocatalytic C=N coupling from H_2CO and NO_2^- over the Fe-MoS_2 from 1000 to 3000 cm^{-1}.

According to the in-situ IR spectra, the *NO_2^- (1117 and 1410 cm^{-1}) and *H_2CO (2749 cm^{-1}) are consumed as the applied potentials increase.[13-14] In the meantime, intermediate *NH_2OH is generated on Fe-MoS_2 and accumulated on the surface.[15-16] Subsequently, the desired C=N containing product emerges with characteristic bands at C=N stretching (1664 cm^{-1}), CH_2 scissoring (1504 cm^{-1}), O–H bending (1296 cm^{-1}, multiplet), and CH_2 rocking (1195 cm^{-1}).[17] In addition, *NH_4^+ emerges at more negative potential with a band at 1617 cm^{-1}.[18] The observed spectral evolution thus captures the complete process from reactant adsorption through intermediate formation to final product generation, alongside a competing reduction pathway.

SI Note 21. Scope Extension on $Fe-MoS_2$ in the Flow-cell

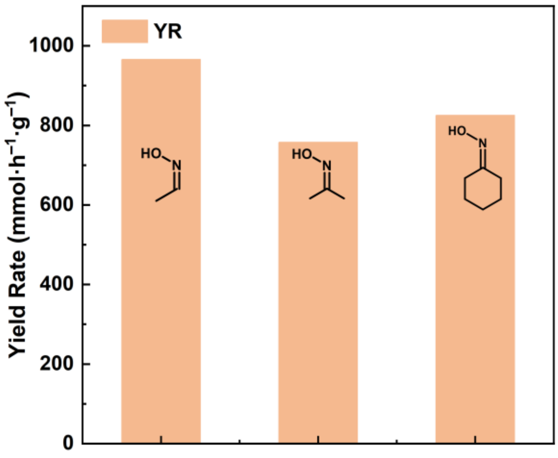

Figure S35. YR for acetaldoxime, 2-propanone oxime, and cyclohexanone oxime on $Fe-MoS_2$ in the flow-cell prototype for 1-hour co-electrolysis.

SI Note 22. Techno-Economic Analysis Calculation

A techno-economic analysis (TEA) calculation for C–N coupling formation in the flow-cell prototype. We change the target in TEA section from $H_2C{=}NOH$ to cyclohexanone oxime based on a thorough assessment of market demand and scalability considerations. Cyclohexanone oxime exhibits high stability, resulting in large-scale manufacturing processes. In addition, cyclohexanone oxime serves as a crucial intermediate precursor for large-scale production of Nylon-6, representing a more relevant and impactful target for our scalable electrocatalytic synthesis pathway.[19] The calculation is based on a modified model reported by Sargent et al.[20] The total costs are divided into two aspects: capital costs (electrolyser and separation costs) and operating costs (electricity, product separation, plant operation, and materials costs). Our assumptions are as below.

1. We assume the plant will have a lifetime of 10 years. Separation equipment capital costs will be assumed to be 10 % of the electrolyzer capital costs.

2. Catalyst costs: Fe-MoS_2 is directly synthesized on carbon cloth with a coating amount of 0.1 mg/cm^2. The precursors of Fe-MoS_2 are $Fe(NO_3)_3{\cdot}9H_2O$ ($2.7 per gram) and $(NH_4)_2MoS_4$ ($118.7 per gram) in DMF ($38.7 per litter) which is simultaneously coated on carbon cloth ($0.09 per cm^2).

3. Electricity cost is assumed as $0.25 kWh^{-1}. The total separation costs are assumed to be 30 % of the electricity cost.

4. Operation costs are assumed as 10 % of catalyst costs and separation device costs.

5. Capacity factor is the fraction of time the plant is expected to be operational on any given day and this is assumed to be 0.8. This means the plant will be operational 19.2 hours a day.

6. The plant will convert 200 tonnes of cyclohexanone into 230 tonnes of cyclohexanone oxime per day. We assume that 90% of the starting ketone and nitrite eventually convert into cyclohexanone oxime; this process therefore consumes 222 tonnes of cyclohexanone and 156 tonnes of $NaNO_2$ per day.

7. The total cell operating voltage is 4 V and the total operating current density is 60 mA cm^{-2}.

8. Feedstock costs include cyclohexanone and $NaNO_2$ which are estimated to be $1635 per tonne and $1160 per tonne, respectively. The price of cyclohexanone oxime is estimated to be $7570 per tonne.

Next, we will calculate each component for a typical day of operation.

Material costs

$$Material\ costs = \frac{Cost\ of\ cyclohexanone \times Mass\ of\ cyclohexanone + Cost\ of\ sodium\ nitrite \times Mass\ of\ sodium\ nitrite}{Mass\ of\ cyclohexanone\ oxime}$$

$$= \frac{\$1635\ \times 222 + 1160 \times 156}{230} = \$2365\ per\ tonne\ of\ cyclohexanone\ oxime$$

Electricity costs

We first calculate the total charge required to obtain 230 tonnes of oxime per day.

$$Q = \frac{Mass\ of\ cyclohexanone\ oxime\ \ converted \times F \times N}{Molar\ mass\ of\ cyclohexanone\ oxime \times Faradaic\ efficiency}$$

$$= \frac{230 \times 96485 \times 4}{113 \times 31.2\%} = 2.5 \times 10^{12}\ C$$

Where Q is the total charge, F is the Faraday's constant and N takes the value 4 since the reduction from cyclohexanone and nitrite to cyclohexanone oxime is a four-electron transfer process.

We now calculate the current required to sustain this process, with a capacity factor of 0.8.

$$I = \frac{Q}{Time\ in\ a\ day \times Capacity\ factor} = \frac{2.5 \times 10^{12}}{24 \times 60 \times 60 \times 0.8} = 3.6 \times 10^{7}\ A$$

Where *I* is the current.

The power required to sustain the process can be calculated (Potentials is assumed as 4 V).

$$P = 3.6 \times 10^{7} \times 4 = 1.4 \times 10^{5}\ kW$$

The energy use per day can be calculated as follows:

$$Energy\ use\ per\ day = P \times Time\ in\ a\ day \times Capacity\ factor$$

$$= 1.4 \times 10^{5} \times 24 \times 0.8 = 2.7 \times 10^{6}\ kWh$$

Finally, the electricity cost per day, normalized by the mass of cyclohexanone oxime produced can be calculated:

$$Energy\ use\ per\ day = \frac{Energy\ use\ perday \times Cost\ per\ kWh}{Mass\ of\ cyclohexanone\ oxime\ produced} = \frac{2.7 \times 10^{6} \times 0.25}{230}$$
$$= \$2935\ per\ tonne\ of\ cyclohexanone\ oxime$$

Total Separation costs

$$Separation\ costs = 30\%\ of\ Electricity\ costs = \$2935 \times 0.3 = \$880.5$$

Capital costs

We calculate the area of electrolyser needed based on the current required and operating current density of 0.06 A·cm^{-2}.

$$Area\ of\ electrolyser = \frac{3.6 \times 10^{7}}{0.06} = 6 \times 10^{4}\ m^{2}$$

The coating amount of Fe-MoS_2 is 0.1 mg per cm^2. The catalyst cost per 1 m^2 can be calculated as below:

$$Catalyst\ cost\ = Cost\ of\ precursors \times Catalyst\ required$$
$$+ Cost\ of\ carbon\ cloth \times carbon\ cloth\ size$$

$$= (0.015 \times 118.7 + 0.00175 \times 2.7 + 0.0125 \times 38.7) \times \left(\frac{10000}{12}\right) + 0.09 \times 10000$$

$$= \$2791\ per\ 1\ m^{2}$$

We can calculate the total catalyst cost using the catalyst area above:

$$Total\ cost\ of\ catalyst = 6 \times 10^{4} \times 2791 = \$1.7 \times 10^{8}$$

The capital costs are associated with the gas/liquid separations equipment are assumed to be 10 % of electrolyser cost and is calculated as:

$$Cost\ of\ separations\ equipment = 1.7 \times 10^{8} \times 0.1 = \$1.7\ \times 10^{7}$$

Finally, the capital costs component can be calculated as:

$$Capital\ costs = \frac{Cost\ of\ electrolyser + Cost\ of\ separations\ equipment}{Lifetime\ of\ plant \times Mass\ of\ cyclohexanone\ oxime\ produced}$$

$$= \frac{\$1.7 \times 10^8 + \$1.7 \times 10^7}{10 \times 365 \times 230} = \$223\ per\ tonne\ of\ formaldoxime$$

Operation costs

This is assumed to be 10% of the capital costs

$$Operation\ cost\ per\ day = \$223 \times 0.1 = \$22.3\ per\ tonne\ of\ cyclohexanone\ oxime$$

Total plant gate levelized costs

Finally, the total cost can now be calculated by adding up all 5 components:

$$Total\ costs = \$2365 + \$2935 + \$880.5 + \$223 + \$22.3$$

$$= \$6425.8\ per\ tonne\ of\ cyclohexanone\ oxime$$

Potential profit

The profit per day from this process can be calculated based on the market price of cyclohexanone oxime, which we assume to be $30,000 per tonne.

$$Profit\ per\ tonne\ of\ \text{cyclohexanone oxime} = \$7570 - \$6425.8 = \$1144.2$$

Therefore, total profits of cyclohexanone oxime per day:

$$Profit\ per\ day = \$1144.2 \times 230 = \$236166$$

Supplementary References

[1] J. Li, Y. Zhang, C. Liu, L. Zheng, E. Petit, K. Qi, Y. Zhang, H. Wu, W. Wang, A. Tiberj, X. Wang, M. Chhowalla, L. Lajaunie, R. Yu, D. Voiry, "3.4% Solar-to-Ammonia Efficiency from Nitrate Using Fe Single Atomic Catalyst Supported on MoS_2 Nanosheets" *Adv. Funct. Mater.* **2022**, *32*, 2108316.

[2] Y.-B. Xue, Y.-M. Cao, P. Luo, X.-X. Dong, B.-B. Han, Y.-D. Zhao, M. Zheng, M. Zheng, Z.-S. Wang, M.-P. Zhuo, "Asymmetric Sandwich Janus Structure for High-Performance Textile-Based Thermo–Hydroelectric Generators Toward Human Health Monitoring" *Adv. Funct. Mater.* **2024**, *34*, 2310485.

[3] J. Tian, C. Yang, R. Hao, F. Li, Z. Liu, W. Chen, Y. Lv, C. Lin, "Fabrication of phosphorus-mediated MoS_2 nanosheets on carbon cloth for enhanced hydrogen evolution reaction" *Int. J. Hydrogen Energy* **2022**, *47*, 17871-17878.

[4] S. I. Zabinsky, J. J. Rehr, A. Ankudinov, R. C. Albers, M. J. Eller, "Multiple-scattering calculations of x-ray-absorption spectra" *Phys. Rev. B* **1995**, *52*, 2995-3009.

[5] B. Ravel, M. Newville, "ATHENA, ARTEMIS, HEPHAESTUS: data analysis for X-ray absorption spectroscopy using IFEFFIT" *J. Synchrotron Radiat.* **2005**, *12*, 537-541.

[6] Y. Wu, Z. Jiang, Z. Lin, Y. Liang, H. Wang, "Direct Electrosynthesis of Methylamine from Carbon Dioxide and Nitrate" *Nat. Sustain.* **2021**, *4*, 725-730.

[7] C. E. Bower, T. Holm-Hansen, "A Salicylate–Hypochlorite Method for Determining Ammonia in Seawater" *Can. J. Fish. Aquat.Sci.* **1980**, *37*, 794-798.

[8] C. G. van de Moesdijk, *The Catalytic Reduction of Nitrate and Nitric Oxide to Hydroxylamine: Kinetics and Mechanism*, de auteur, **1979**, p. 56.

[9] P. E. Blöchl, "Projector Augmented-Wave Method" *Phys. Rev. B* **1994**, *50*, 17953-17979.

[10] G. Kresse, J. Furthmüller, "Efficient Iterative Schemes for ab initio Total-Energy Calculations using a Plane-Wave Basis Set" *Phys. Rev. B* **1996**, *54*, 11169-11186.

[11] J. P. Perdew, K. Burke, M. Ernzerhof, "Generalized Gradient Approximation Made Simple" *Phys. Rev. Lett.* **1996**, *77*, 3865-3868.

[12] G. Henkelman, A. Arnaldsson, H. Jónsson, "A Fast and Robust Algorithm for Bader Decomposition of Charge Density" *Comput. Mater. Sci.* **2006**, *36*, 354-360.

[13] Y. Zhao, Y. Ding, W. Li, C. Liu, Y. Li, Z. Zhao, Y. Shan, F. Li, L. Sun, F. Li, "Efficient urea electrosynthesis from carbon dioxide and nitrate via alternating Cu–W bimetallic C–N coupling sites" *Nat. Commun.* **2023**, *14*, 4491.

[14] K. Z. Gaca-Zając, B. R. Smith, A. Nordon, A. J. Fletcher, K. Johnston, J. Sefcik, "Investigation of IR and Raman spectra of species present in formaldehyde-water-methanol systems" *Vib. Spectrosc.* **2018**, *97*, 44-54.

[15] W. Chen, Y. Wu, Y. Jiang, G. Yang, Y. Li, L. Xu, M. Yang, B. Wu, Y. Pan, Y. Xu, Q. Liu, C. Chen, F. Peng, S. Wang, Y. Zou, "Catalyst Selection over an Electrochemical Reductive Coupling Reaction toward Direct Electrosynthesis

of Oxime from NOx and Aldehyde" *J. Am. Chem. Soc.* **2024**, *146*, 6294-6306.

[16] M. Li, Y. Wu, B.-H. Zhao, C. Cheng, J. Zhao, C. Liu, B. Zhang, "Electrosynthesis of amino acids from NO and α-keto acids using two decoupled flow reactors" *Nat. Catal.* **2023**, *6*, 906-915.

[17] Q. Y. Wu, T. L. Tan, "Fourier transform infrared (FTIR) spectroscopy of formaldoxime (CH_2NOH) in the 450–3800 cm^{-1} region and its ν9 band" *J. Mol. Spectrosc.* **2021**, *376*, 111417.

[18] Z. Tao, C. L. Rooney, Y. Liang, H. Wang, "Accessing Organonitrogen Compounds via C–N Coupling in Electrocatalytic CO_2 Reduction" *J. Am. Chem. Soc.* **2021**, *143*, 19630-19642.

[19] J. Sharp, A. Ciotti, H. Andrews, S. R. Udayasurian, M. García-Melchor, T. Li, "Sustainable Electrosynthesis of Cyclohexanone Oxime through Nitrate Reduction on a Zn–Cu Alloy Catalyst" *ACS Catal.* **2024**, *14*, 3287-3297.

[20] Y. Lum, J. E. Huang, Z. Wang, M. Luo, D.-H. Nam, W. R. Leow, B. Chen, J. Wicks, Y. C. Li, Y. Wang, C.-T. Dinh, J. Li, T.-T. Zhuang, F. Li, T.-K. Sham, D. Sinton, E. H. Sargent, "Tuning OH Binding Energy Enables Selective Electrochemical Oxidation of Ethylene to Ethylene Glycol" *Nat. Catal.* **2020**, *3*, 14-22.
